# Supplementary material for: Insight in the development of the mutual trust relationship between trainers and trainees in a workplace-based postgraduate medical training programme: a focus group study among trainers and trainees of the Dutch general practice training programme
Source: BMJ Open. 2020 Apr 19;10(4):e036593. doi: 10.1136/bmjopen-2019-036593 (PMC7245404; doi:10.1136/bmjopen-2019-036593)
Supplement: Author's manuscript [file bmjopen-2019-036593.draft_revisions.pdf]

BMJ Open

BMJ Open is committed to open peer review. As part of this commitment we make the peer review history of every article we publish publicly available.

When an article is published we post the peer reviewers' comments and the authors' responses online. We also post the versions of the paper that were used during peer review. These are the versions that the peer review comments apply to.

The versions of the paper that follow are the versions that were submitted during the peer review process. They are not the versions of record or the final published versions. They should not be cited or distributed as the published version of this manuscript.

BMJ Open is an open access journal and the full, final, typeset and author-corrected version of record of the manuscript is available on our site with no access controls, subscription charges or pay-per-view fees (http://bmjopen.bmj.com).

If you have any questions on BMJ Open's open peer review process please email info.bmjopen@bmj.com

BMJ Open

Development of the mutual trust relationship between trainers and trainees in workplace-based medical training: results of a focus group study.

Journal:	*BMJ Open*
Manuscript ID	bmjopen-2019-036593
Article Type:	Original research
Date Submitted by the Author:	02-Jan-2020
Complete List of Authors:	Bonnie, Linda; Amsterdam UMC - Locatie AMC, General Practice<br>Visser, Mechteld; Amsterdam UMC - Locatie AMC, Department of General Practice/GP Specialty Training<br>Kramer, Anneke ; Leiden University<br>van Dijk, Nynke; Amsterdam UMC - Locatie AMC
Keywords:	MEDICAL EDUCATION & TRAINING, QUALITATIVE RESEARCH, PRIMARY CARE

SCHOLARONE™
Manuscripts

I, the Submitting Author has the right to grant and does grant on behalf of all authors of the Work (as defined in the below author licence), an exclusive licence and/or a non-exclusive licence for contributions from authors who are: i) UK Crown employees; ii) where BMJ has agreed a CC-BY licence shall apply, and/or iii) in accordance with the terms applicable for US Federal Government officers or employees acting as part of their official duties; on a worldwide, perpetual, irrevocable, royalty-free basis to BMJ Publishing Group Ltd ("BMJ") its licensees and where the relevant Journal is co-owned by BMJ to the co-owners of the Journal, to publish the Work in this journal and any other BMJ products and to exploit all rights, as set out in our licence.

The Submitting Author accepts and understands that any supply made under these terms is made by BMJ to the Submitting Author unless you are acting as an employee on behalf of your employer or a postgraduate student of an affiliated institution which is paying any applicable article publishing charge ("APC") for Open Access articles. Where the Submitting Author wishes to make the Work available on an Open Access basis (and intends to pay the relevant APC), the terms of reuse of such Open Access shall be governed by a Creative Commons licence – details of these licences and which Creative Commons licence will apply to this Work are set out in our licence referred to above.

Other than as permitted in any relevant BMJ Author's Self Archiving Policies, I confirm this Work has not been accepted for publication elsewhere, is not being considered for publication elsewhere and does not duplicate material already published. I confirm all authors consent to publication of this Work and authorise the granting of this licence.

For peer review only

Development of the mutual trust relationship between trainers and trainees in workplace-based medical training: results of a focus group study.

Authors

Linda H.A. Bonnie[a] (Msc MD), Mechteld R.M. Visser[a] (PhD), Anneke W.M. Kramer[b] (Professor, MD, PhD), Nynke van Dijk[a] (Professor, MD, PhD)

Affiliations

[a] Amsterdam UMC, Academic Medical Center; Department of General Practice; Amsterdam Public Health Research Institute; Amsterdam, The Netherlands

[b] Leiden University Medical Center; Department of Public Health and Primary Care; Leiden, The Netherlands

Corresponding author

Linda H.A. Bonnie, MsC MD

ORCID iD: 0000-0002-5581-8761

Institution: Amsterdam UMC, University of Amsterdam

Department: Department of General Practice, Amsterdam Public Health research institute

Adress: Meibergdreef 9, 1105 AZ Amsterdam, The Netherlands

Telephone number: 0031-20-5667165

E-mail address: l.h.bonnie@amsterdamumc.nl

For peer review only

Abstract

Objectives

Trust plays an important role in workplace-based postgraduate medical education (PGME) programmes. Trainers must trust their trainees for granting them greater independence. Trainees must trust their trainer for a safe learning environment. As trainers' and trainees' trust in each other plays an important role in trainee learning and development, the authors aimed to explore the development of the mutual trust relationship between trainers and trainees.

Setting

This study was performed in a General Practice training department in the Netherlands.

Participants

All trainers and trainees of the General Practice training department were invited to participate. Fifteen trainers and 34 trainees, voluntarily participated in focus group discussions.

Outcome measures

The authors aimed to gain insight in the factors involved in the development of the mutual trust relationship between trainers and trainees, in order to be able to create a model for the development of a mutual trust relationship between trainers and trainees.

Results

For peer review only

For peer review only - http://bmjopen.bmj.com/site/about/guidelines.xhtml

In the first stage of trust development, trainers and trainees develop basic trust in each other. Basic trust forms the foundation of the trust relationship. In the second stage, trainers develop trust in trainees taking into account trainees' working and learning performance, and the context in which the work is performed. Trainees trust their trainer based on the trainer's availability and accessibility and the personal relationship between the trainee and their trainer. Trainee self-confidence modifies the development of a trust relationship.

Conclusion

The development of a mutual trust relationship between trainers and trainees is a complex process that involves various stages, goals, factors, and interactive aspects. As the mutual trust relationship influences the learning environment for trainees, greater emphasis on the mutual trust relationship may improve learning outcomes. Further research may explore the effect of long-term and short-term educational relationships on the trust relationship between trainers and trainees.

For peer review only

For peer review only - http://bmjopen.bmj.com/site/about/guidelines.xhtml

Article Summary

Strengths and limitations of this study

- We were able to include a relatively large amount of trainers and trainees, in various stages of the training programme, which provided us with insights in many different experiences and viewpoints about the trust relationship between trainers and trainees.

- This study is a single-centre study, focussing on the development of a trust relationship in the GP training programme, which may cause results to be not directly applicable in other PGME programs.

- Trainees provided us with a number of important insights concerning the effect of an educational relationship's duration on the trust relationship between trainers and trainees, we did however not explore the vision of trainers involved in short rotations.

For peer review only - http://bmjopen.bmj.com/site/about/guidelines.xhtml

Funding

This publication was written as a part of the project “The use of Entrustable Professional Activities in Assessment in General Practice Specialty Training” (project number 839130004), that has received fundings from the “Netherlands Organisation for Health Research and Development” (ZonMW).

Conflicts of interests

L.H.A. Bonnie, M.R.M. Visser, A.W.M. Kramer, and N. van Dijk declare that they have no conflicts of interest. The authors alone are responsible for the content and the writing of this article.

For peer review only

For peer review only - http://bmjopen.bmj.com/site/about/guidelines.xhtml

Introduction

Trust is fundamental to workplace-based learning in post graduate medical education (PGME) programmes.(1) In workplace-based PGME programmes, trainees learn to work independently when providing patient care, with the goal of becoming independently functioning medical specialists.(2, 3) In the process of learning to work independently, trainees are supervised by trainers, who are educators for trainees, next to their work as patient care providers.(4) In the process of supervision, trainers must balance the freedom they give their trainees in independently providing patient care, against patient safety. Trainers thus have to trust their trainee to provide good quality of care for their patients.(1, 5, 6) In this process of trust development, trainers take into account factors that are related to themselves as a trainer and as a patient care provider, the trainee, the trainer-trainee relationship, the context in which the work is performed and the nature of the task in question.(1, 6-11) Once trainers trust their trainee, they can gradually entrust them with performing patient care independently.(1)

Trust is not just important in terms of patient-safety, it is also a prerequisite for successful trainee learning. Being trusted by a trainer allows trainees to increasingly learn to work independently in the workplace and thereby develop themselves towards becoming independently functioning medical specialists.(1) Additionally, trainers entrusting trainees to perform tasks independently can boost motivation for learning and working.(1, 7) However, for trainees being able to learn from experiences in the workplace, to perform their work properly, and being able to receive feedback, a safe learning environment is required. Trusting their trainer is essential for trainees to experience a safe learning environment.(12-16)

For peer review only

For peer review only - http://bmjopen.bmj.com/site/about/guidelines.xhtml

112 However, factors involved in the process of trainees developing trust in their trainer still are
113 poorly understood.

115 As both trainer's trust in their trainee(1), as well as trainee's trust in their trainer(12-16), play
116 an important role in trainee learning and development, understanding the mutual trust
117 relationship might help to optimize the learning outcomes for trainees.(12, 13) As factors
118 involved in the process of trust development of trainees in their trainer still remain poorly
119 understood, and little information is available about how the trust development processes of
120 both trainers and trainees may influence each other, we aim to gain insight in the development
121 of the mutual trust relationship between trainers and trainees in a workplace-based PGME-
122 programme.

For peer review only - http://bmjopen.bmj.com/site/about/guidelines.xhtml

Methods

Context

This focus group study was conducted among the trainers and trainees of a Dutch GP-training programme. Dutch GPs are a pivotal element of the Dutch health-care system. In the event of a medical problem (life-threatening situations excluded), all Dutch inhabitants first visit their GP for advice and treatment. If necessary the GP refers to a medical specialist, for further evaluation.(17, 18) To prepare trainee GPs for this broad field of work, the Dutch GP-training program involves a significant amount of workplace-based learning. The first and third year of the training programme are spent working in general practice. During this period, trainees are supervised by one or two specially trained GPs, who will be coaching, teaching and assessing the trainee. The second year of their training programme is dedicated to traineeships in the areas of clinical or emergency medicine, care for the elderly and psychiatry. The trainers in these internships are medical specialists who have been trained to supervise trainee GPs.(19, 20)

Study design

As we aimed to explore the process of development of the mutual trust relationship between trainers and trainees in a workplace-based PGME-program, we adopted a qualitative approach. We chose to use focus group discussion with trainers and trainees for data collection, which we believed would provide insight in participants' experiences and views regarding the development of trust. Individual differences in experience and views could be shared and explored in interactive group discussions. (21, 22)

For peer review only - http://bmjopen.bmj.com/site/about/guidelines.xhtml

For the analysis of the research data, we used thematic analysis, as this is a method for "identifying, analysing, and reporting patterns within data".(23) With the thematic analysis we aimed to gain insight in the process of development of the mutual trust relationship between trainers and trainees. Although an extensive amount of literature is available about trainers developing trust in their trainees(1, 6-11), we chose to apply an inductive approach for the coding and analysis of all research data, as we hoped to identify factors involved in the development of the mutual trust relationship between trainers and trainees that currently are poorly understood an not yet described in the literature.(23, 24)

Participants

We conducted this study among GP trainers and trainee GPs from the Amsterdam UMC – location AMC GP training institute. We selected homogeneous groups, i.e. separate groups for trainers and trainees, as we expected that discussion of some aspects of trust could place participants in a vulnerable position. It was hoped that eliminating any hierarchy in the focus groups would promote free and open discussions.(21)

All trainers and trainees from the Amsterdam UMC – location AMC GP training institute were informed about the purpose of the study, in person and by email, by the principle researcher (LB). Focus group meetings for trainers were held during the faculty development programme. Trainers could voluntary sign up for participation in the focus group discussions, after having received information about the study. Trainees participated in focus groups during their weekly educational day, they could voluntarily sign up for participation in the focus group discussions after information about the study was provided to them.

Data collection

For peer review only - http://bmjopen.bmj.com/site/about/guidelines.xhtml

The data used in this study was obtained from a larger study into the implementation of Entrustable Professional Activities (EPAs) in the GP-training programme. The practical aspects of the use of EPAs in the GP-training programme are presented elsewhere.(25) Between November 2016 and March 2017, there were two trainer focus group discussions and four trainee focus group discussions. Each focus group consisted of six to eleven participants, and lasted for 45-70 minutes. Demographic data (age, gender and length of career to date) on the participants was collected by means of a questionnaire.

Each focus group discussion was led by a skilled moderator (MV or NvD), while an assistant-moderator (LB or a research assistant) took notes.

The sessions started with a presentation on the purpose of the study, during which participants had the opportunity to ask questions. An exploratory question initiated the discussion, after which key questions were used to maintain the momentum. The topics covered (Table 1) were drawn from the literature on trust in workplace-based medical education.(1, 8, 10, 26) Data collection and data analysis were performed iteratively. The results of the data analysis were used to guide subsequent data collection.(21) The outcomes of the focus group discussions of both trainers and trainees were used to modify the topic list used in following focus group discussions with both trainers and trainees, making it possible to explore trainers' and trainees' views and practical experience of trust in broader detail.

After each meeting, the moderator and assistant-moderator conducted a debriefing to discuss the course and the details of the discussion. The debriefing notes formed part of the dataset.

Data analysis

For peer review only - http://bmjopen.bmj.com/site/about/guidelines.xhtml

Audio recordings of the discussions were transcribed verbatim and anonymized. In the first step, LB (who is a GP-trainee) performed an inductive coding process in four focus group discussions. The first step in the coding process resulted in a code book with themes that emerged from the focus group discussions. The details of this code book were discussed at length with NvD (who is a MD and an educationalist), to ensure coding reliability. In the second step, LB and NvD performed an axial coding process involving the creation of overarching categories in the code book. In the third step, LB encoded the remaining two focus groups, using the code book derived from the axial coding process. As no new themes emerged from the last two focus groups, so we assumed that data saturation had been reached. Once again, the code book was discussed in detail with NvD. The fourth step in the process of data analysis involved selective coding of the data. NvD, MV (who is a psychologist) and LB used the axial-coded code book to identify any themes that were relevant to the research question and to identify any relationships between the relevant themes. This process was supported by the use of memos and diagrams.(23, 27, 28) In the case of uncertainties or disagreement between the researchers during the coding process, the verbatim transcripts and audio-recorded data were consulted.

We used MAXQDA 2018 (VERBI Software (2017), Berlin, Germany) for data analysis.

Ethical approval

This study was approved by the Ethical Review Board of the Netherlands Association for Medical Education (ERB-NVMO, file-number 664). The participants were fully informed about the purpose of the study, participation was entirely voluntary, and individuals were able to withdraw at any time without having to give a reason. Prior to the focus group discussions,

For peer review only - http://bmjopen.bmj.com/site/about/guidelines.xhtml

For peer review only

participants signed an informed consent form. All data was collected, stored and processed anonymously. No rewards were offered for participation in the study.

Patient and public involvement

There were no patients involved in this study.

For peer review only

For peer review only - http://bmjopen.bmj.com/site/about/guidelines.xhtml

Results

Fifteen GP trainers and 34 trainee GPs participated in the study. Participant characteristics are displayed in Table 2. Overall, the atmosphere during the focus groups was pleasant. Participants felt safe to share their experiences, even though some experiences led to emotional reactions.

Based on our data we created a model for the development of a trust relationship between trainers and trainees (Figure 1). The development of trust involves multiple factors and stages, and also shows some interactive aspects. Trust development starts with the first impression, based on which both trainers and trainees develop a level of basic trust in one another. Further development of the trust relationship between trainers and trainees during the course of the training programme is related to the process of training the trainee. Trainers and trainees adopt different approaches to building trust and have different goals. Trainers base the trust in their trainee on the performance of the trainee in working and learning in daily clinical practice, while trainees focus on the personal relationship with their trainer. Trainee self-confidence is a major factor affecting the development of the trust relationship in both directions. Below we further describe the components of the development of trust.

Stage 1: Basic trust

Based on their first impressions, trainers and trainees each start to develop basic trust in the other. Basic trust is not typically related to the training of the trainee, but entails a more generic kind of trust in the other person. Basic trust forms the foundation of the trust relationship between trainers and trainees. The process of developing basic trust seems difficult to put into words. Both trainers and trainees refer to it as a “feeling”, or “click” that

For peer review only - http://bmjopen.bmj.com/site/about/guidelines.xhtml

they experience with the other person. They mention that the way of communicating with each other and a relaxed atmosphere during the first encounter play an important role in this process. Additionally, matching personality traits and a feeling of mutual understanding, also support the trust development process in this first short period.

Quote (trainee GP, 2nd year): "The development of trust starts with a good first impression. But it's not good if there is no 'click'."

Both trainers and trainees indicate that first impressions have a strong influence on their trust in each other. Once established, the trust relationship tends to be difficult to adjust, also when factors occur that might influence the degree of trust in the other person.

Quote (GP trainer): "There is very little scope for modifying a first impression. Your impression of the trainee turns out to be virtually immutable."

For peer review only

Stage 2: Trust development related to the training of the trainee

Once trainers and trainees start working together in daily clinical practice, the focus of the trust relationship shifts towards the process of training the trainee, based on experiences during working and learning in daily clinical practice. Trainers and trainees have different approaches to and different goals for building a trust relationship, but joint conditions for trust development are also present.

The development of a trainer's trust in their trainee

Trainers have to develop trust in their trainee in order to be able to entrust them to see patients independently. This trust is based on factors such as the trainee's performance

For peer review only - http://bmjopen.bmj.com/site/about/guidelines.xhtml

in working and learning, context-dependent factors, and the degree of respect trainees show to patients, trainers and practice staff.

Trainee performance in working and learning is reflected in the trainee's transparency, their openness about how they perform their work, and their ability to learn from previous feedback. Trainers tend to focus on how trainees communicate about their positive and negative (learning) experiences in the workplace, on how they deal with trainer feedback, whether they know their own limitations, and ask for help at the right moments.

Quote (GP trainer): " Trust is also based on the trainee's openness about discussing past cases with me. If all I hear from them are success stories, while my assistant or patients give me conflicting reports, that gets me thinking: 'Wait a moment…'"

Quote (GP trainer): "We do not blindly trust trainees, believing them to be capable of dealing with any and all situations. We trust them because we know that, when they find themselves in situations of which they have no previous experience, they will be able to deal with them. They are aware of their own limitations, and they know when to ask for help."

Context-dependent factors also influence the degree of trust that trainers place in their trainees. These include the nature of the patient's problem, and (special) situations in which the patient is presented. Trust is put to the test in emergency consultations, during house calls or during shifts at the Out-of-Hours GP Centre, when there is a greater risk of serious illness.

For peer review only - http://bmjopen.bmj.com/site/about/guidelines.xhtml

Quote (GP trainer): "Emergency care is typically a topic in which, as a trainer, I need to trust my trainee."

Quote (GP trainer): "In certain areas I think trainees can't do any harm, so I let them try to work independently."

<u>The development of a trainee's trust in their trainer</u>

Trainees have to trust their trainer in order to experience a safe learning environment. This trust is based on the trainer's availability and accessibility, and on the trainer's ability to create an environment in which the trainee feels that the trainer is willing to listen to them and help them. If trainers are accessible, wherever and whenever required, this gives trainees a feeling of security, enhancing their trust in their trainer.

Quote (trainee GP, 2nd year): "I think it also has to do with accessibility. My GP trainer is very accessible, which increases my trust in him considerably."

Also, if trainers show a personal interest in their trainee, this creates a feeling of connectedness which, in turn, promotes the development of trust. Trainees find it more easy to be open about their performance and learning in daily clinical practice when they feel safe with, and connected to their trainer, especially in the case of problems and negative experiences.

Quote (trainee GP, 2nd year): "I did not have a good relationship with my trainer, there was no real click at the personal level, which made it harder for us to work together. It also made it harder for me to show my vulnerabilities…"

For peer review only - http://bmjopen.bmj.com/site/about/guidelines.xhtml

The trainees noticed that, during short, non-GP, rotations (three to six months), where several different trainers are involved in the training of the trainee, their personal relationship with their trainer was less intense than during year-long rotations in the GP-practice, with a maximum of two trainers. During short rotations, and in the case of multiple (more than two) trainers, trainees find it harder to develop a personal connection with, and trust in their trainers.

Quote (trainee GP, 2nd year): "In a whole year in training, you encounter all sorts of problems. It's good to discuss those problems with your trainer. If the individual relationship were less intense, then I would not discuss my personal problems. On short rotations I notice that there is much less of a personal connection."

<u>Joint conditions for trust development</u>

<u>Integrity</u> - Both trainers and trainees build trust based on the integrity of the other person. Trainers and trainees assess each other's integrity in terms of consistent behaviour. They also compare each other's actions with their own work in daily practice and with their own moral standards. Consistent behaviour, and converging working styles and moral standards tend to boost their trust in each other.

Quote (GP trainer): "Sometimes, I don't trust a given trainee, but I don't know why. Usually, it has something to do with their Professional role. Sometimes I think, 'I would not approach someone in that way, I do not want my patients to be treated like that.' Yet, the trainee has done nothing wrong in terms of their Medical Expert role."
Quote (trainee GP, 2nd year): "I think, in fact, that it's easier to trust someone whose approach to work matches your own."

For peer review only - http://bmjopen.bmj.com/site/about/guidelines.xhtml

Trainee self-confidence - Trainee self-confidence is a major factor in the development of trust between trainers and trainees. If trainers entrust trainees to work independently in daily practice, this will improve trainee self-confidence. The effect is enhanced if trainers view trainees as equals and are willing to share their daily responsibilities with them.

If trainers and trainees have the same expectations of the trainee's performance, this boosts trainee self-confidence. However, if a trainee's degree of self-confidence deviates from their trainer's expectations this can impair the trainer's trust, as trainers feel that too much or too little self-confidence could impact patient safety. Such mismatches between the trainer's expectations and the trainee's actual level of self-confidence can also impact the trainee's trust in their trainer, as in those cases trainees feel their trainer is not giving them sufficient support at the level where trainees feel they need it. As trainees are experiencing a lack of support from their trainer, this process will also negatively influence the self-confidence of the trainee.

Quote (GP trainer): "Some trainees tend to overestimate their abilities, and your immediate reaction is that you don't want to entrust your patients to them."

Quote (trainee GP, 1st year): "I get very uncomfortable if my trainer shows me a great deal of trust, because I wonder if I really deserve it."

For peer review only - http://bmjopen.bmj.com/site/about/guidelines.xhtml

Discussion

This study indicates that the process of mutual trust development between trainers and trainees in a workplace-based PGME-programme involves various stages, factors and interactive aspects. The process of mutual trust development starts at the first encounter, with the first impression forming basic trust. Once trainers and trainees start working together in daily clinical practice, the focus of the trust development process shifts towards working and learning in the workplace. In this phase, trainers develop trust in their trainees in order to be able to entrust them with patient care. Trainees need to trust their trainer in order to experience a safe learning environment. Next to trainer- and trainee-related factors, joint conditions for mutual trust development, like integrity and trainee self-confidence, are also involved in the mutual trust development process.

The process of developing a trust relationship between two people knows various stages. In the first stage, the involved persons have to get to know each other. Once they have become familiar with each other, the trust development process will increasingly be based on the knowledge about the other person and about the relationship that they have developed.(30) Tschannen-Moran and Hoy(31) assume that learners follow four stages in developing trust in their teachers: the initial impression phase, the exploration phase, the limit testing phase, and the stability phase.(31, 32) The findings of our study mainly reflect the initial impression stage (basic trust development) and the exploration and limit-testing stage (trust development focusing on working and learning in daily practice). In our study, the stage of development of basic trust (initial impression stage), seems to have a strong influence on further trust development process. This is in line with other studies saying that the amount of trust

For peer review only - http://bmjopen.bmj.com/site/about/guidelines.xhtml

397 developed in the initial stage influences the likelihood to see factors and situations that further
398 develop trust.(33)
399
400 In the second stage of trust development, when the trust development process focusses on
401 working and learning in daily practice, trainers and trainees have different styles in trusting
402 each other, and different goals for trusting each other. The factors influencing the trust of
403 trainers in their trainees were the same ones identified by Hauer, et al. (10), and Sagasser, et
404 al. (11) in their study into this process. Trainees in the second stage of trust development
405 develop trust in their trainer based on the availability and accessibility of the trainer for the
406 trainee, and on their personal relationship. This is in line with factors that Sutkin et al.
407 identified as students' criteria for good clinical teachers(34), and with earlier findings that a
408 caring attitude towards learners may support trust development from trainees in their trainer.
409 (31, 35) This might explain why trainees, more than trainers, value the personal relationship
410 when developing trust in their trainer.
411
412 The trainees noticed that the trust relationship with their trainer was stronger during long
413 rotations (a minimum of one year) than it was during short rotations (usually three to six
414 months), especially due to the fact that they were able to develop a more intense personal
415 relationship with their trainer. Hirsh et al. found that this effect may be due to the more
416 effective distribution of time during longitudinal rotations.(36) A more intense personal
417 relationship during long rotations encourages trainees to discuss their performance with their
418 trainer more often, leading to greater shared responsibility in identifying approaches to deal
419 with any weaknesses in the trainee's performance. It also helped trainees to take a more
420 constructive view of their trainer's feedback and to provide better patient care.(37)

For peer review only - http://bmjopen.bmj.com/site/about/guidelines.xhtml

Longitudinal rotations may therefore not only lead to an improved learning environment with more learning opportunities for the trainee, it might also help to improve patient care.

Strengths and limitations

For this study we were able to include a relatively large amount of trainers and trainees, in various stages of the training programme, which provided us with insights in many different experiences and viewpoints about the trust relationship between trainers and trainees.

This study is a single-centre study, focussing on the development of a trust relationship in the GP training programme. Therefore, the results might not be directly applicable in other PGME programs. We were however able to identify factors that might influence the trust development process in (non-GP) short rotations or rotations with multiple trainers. We therefore think that aspects of our study may help various kinds of PGME programmes to further optimize the learning environment for their trainees.

The trainees provided us with a number of important insights concerning the effect of an educational relationship's duration on the trust relationship between trainers and trainees. However, in this study we did not explore the vision of trainers involved in short rotations. The distinction between long-term and short-term educational relationships and their effect on the trust relationships between trainers and trainees were therefore not fully explored and merit further study.

Implications for practice

During the first impression, the foundation is made for the trust relationship between trainers and trainees. By introducing trainers and trainees to each other before the start of a training

For peer review only - http://bmjopen.bmj.com/site/about/guidelines.xhtml

period, a solid foundation for the trust relationship can be established. When trainers and trainees are aware of the halo-effect of trust and of the differences in their needs concerning the trust relationship, the trust relationship can be enhanced.

Furthermore, a long duration of a training relationship positively influences the trust relationship between trainers and trainees when compared to short-term training relationships, which might improve trainee performance and patient care.(37, 38) When long-term training relationships are difficult to realize, the findings of this study might help trainers in short-term training relationships to improve the trust relationship between trainers and trainees, which could also improve the learning environment for trainees and patient care.

For peer review only - http://bmjopen.bmj.com/site/about/guidelines.xhtml

Figures

Figure 1: Model for the mutual trust relationship between trainers and trainees.

For peer review only

For peer review only - http://bmjopen.bmj.com/site/about/guidelines.xhtml

Acknowledgements

The authors wish to thank the participating trainers and trainees for their willingness to contribute to the study.

Author's contribution

Linda H.A. Bonnie: Substantial contribution to the conception and design of the work, and to the acquisition, analysis and interpretation of data. Substantial contribution in drafting the work. Provides final approval of the version to be published. Agrees to be accountable for all aspects of the work.

Mechteld R.M. Visser: Substantial contribution to the acquisition, analysis and interpretation of data. Substantial contribution in critically revising the work. Provides final approval of the version to be published. Agrees to be accountable for all aspects of the work.

Anna W.M. Kramer: Substantial contribution to the conception of the work and the interpretation of data. Substantial contribution in critically revising the work. Provides final approval of the version to be published. Agrees to be accountable for all aspects of the work.

Nynke van Dijk: Substantial contribution to the conception and design of the work, and to the acquisition, analysis and interpretation of data. Substantial contribution in drafting and critically revising the work. Provides final approval of the version to be published. Agrees to be accountable for all aspects of the work.

Data sharing

The data for this paper may be obtained from the authors upon request.

For peer review only - http://bmjopen.bmj.com/site/about/guidelines.xhtml

Word count

Abstract: 300

Main text: 3987

Number of figures: 1

Number of tables: 2

For peer review only

For peer review only - http://bmjopen.bmj.com/site/about/guidelines.xhtml

Literature

1. Hauer KE, Ten Cate O, Boscardin C, Irby DM, Iobst W, O'Sullivan PS. Understanding trust as an essential element of trainee supervision and learning in the workplace. Adv Health Sci Educ Theory Pract. 2014;19(3):435-56.

2. Kennedy TJ, Regehr G, Baker GR, Lingard LA. Progressive independence in clinical training: a tradition worth defending? Academic medicine : journal of the Association of American Medical Colleges. 2005;80:S106-S11.

3. Dornan T, Boshuizen H, King N, Scherpbier A. Experience-based learning: a model linking the processes and outcomes of medical students' workplace learning. Med Educ. 2007;41(1):84-91.

4. Taylor EW, Tisdell EJ, Gusic ME. Teaching beliefs of medical educators: perspectives on clinical teaching in pediatrics. Medical teacher. 2007;29:371-6.

5. Carracio C, Englander R, Holmboe ES, Kogan JR. Driving care quality: Aligning trainee assessment and supervision through practical application of entrustable professional activities, competencies, and milestones. . Academic medicine : journal of the Association of American Medical Colleges. 2016;91:199-203.

6. ten Cate O, Scheele F. Viewpoint: competency-based postgraduate training: can we bridge the gap between theory and clinical practice? Academic medicine : journal of the Association of American Medical Colleges. 2007;82(6):542-7.

7. Sterkenburg A, Barach P, Kalkman C, Gielen M, ten Cate O. When do supervising physicians decide to entrust residents with unsupervised tasks? Academic medicine : journal of the Association of American Medical Colleges. 2010;85(9):1408-17.

For peer review only - http://bmjopen.bmj.com/site/about/guidelines.xhtml

8. Choo KJ, Arora VM, Barach P, Johnson JK, Farnan JM. How do supervising physicians decide to entrust residents with unsupervised tasks? A qualitative analysis. J Hosp Med. 2014;9(3):169-75.

9. Dijksterhuis MG, Voorhuis M, Teunissen PW, Schuwirth LW, ten Cate OT, Braat DD, et al. Assessment of competence and progressive independence in postgraduate clinical training. Med Educ. 2009;43(12):1156-65.

10. Hauer KE, Oza SK, Kogan JR, Stankiewicz CA, Stenfors-Hayes T, Cate OT, et al. How clinical supervisors develop trust in their trainees: a qualitative study. Med Educ. 2015;49(8):783-95.

11. Sagasser MH, Fluit CRMG, van Weel C, van der Vleuten CPM, Kramer AWM. How entrustment is informed by holistic judgements across time in a family medicne residency program: an ethnographic nonparticipant observational study. Academic medicine : journal of the Association of American Medical Colleges. Epub ahead of print.

12. Frandsen Gran S, A.M. B, Lindbaek M, Frich JC. General practicioners'and students'experiences with feedback during a six-week clerkship in general practice: a qualitative study. Scand J Prim Health Care. 2016;34(2):171-8.

13. Sklar DP. Trust is a two-way street. Academic medicine : journal of the Association of American Medical Colleges. 2016;91:155-8.

14. Carless D. Trust, distrust and their impact on assessment reform. Assessment & Evaluation in Higher Education. 2009;34(1):79-89.

15. Evans C. Making sense of assessment feedback in higher education. Rev Educ Res. 2013;83(1):70-120.

16. Epstein RM. Assessment in medical education. N Engl J Med. 2007;356(4):387-96.

For peer review only - http://bmjopen.bmj.com/site/about/guidelines.xhtml

17. Publiek Z. General Practice care (Available in Dutch: Huisartsenzorg) 2015 [Accessed March 2016]. Available from: https://www.zorgprismapubliek.nl/informatie-over/huisartsenzorg/huisartsenzorg/.

18. van Weel C. Primary health care and family medicine at the core of health care: challanges and priorities in how to further strengthen their potential. Front Med. 2014;1(37):1-5.

19. Huisartsopleiding Nederland. What does the training program look like? (Available in Dutch: Hoe ziet de opleiding eruit?) [Accessed 09-12-2015]. Available from: http://uitdepraktijk.huisartsopleiding.nl/opleiding/hoe-ziet-de-opleiding-eruit.

20. College voor Huisartsgeneeskunde, Verpleeghuisgeneeskunde en medische zorg voor verstandelijk gehandicapten. Decree on general practice medicine regarding registration requirements for general practitioners. (Available in Dutch: Besluit huisartsgeneeskunde; Besluit van 21 augustus 2008 houdende opleidings, erkennings- en (her)registratie-eisen voor het specialisme huisartsgeneeskunde), (2015).

21. Stalmeijer RE, McNaughton N, Van Mook WN. Using focus groups in medical education research: AMEE Guide No. 91. Medical teacher. 2014;36(11):923-39.

22. Kitzinger J. Qualitative research. Introducing focus groups. BMJ. 1995;311(7000):299.

23. Braun V, Clarke V. Using thematic analysis in psychology. Qual Res Psychol. 2006;3(2):77-101.

24. Braun V, Clarke V. Thematic analysis. In: Cooper H, editor. APA handbook of research methods in psychology. 2. Research designs: American Psychological Association; 2012. p. 57-70.

For peer review only

For peer review only - http://bmjopen.bmj.com/site/about/guidelines.xhtml

563 25. Bonnie LHA, Visser MRM, Bont J, Kramer AWM, van Dijk N. Trainers' and Trainees' expectations of Entrustable Professional Activities (EPAs) in a Primary Care training programme. Education for Primary Care. 2019;30(1):13-21.

566 26. ten Cate O, Hart D, Ankel F, Busari J, Englander R, Glasgow N, et al. Entrustment Decision Making in Clinical Training. Academic medicine : journal of the Association of American Medical Colleges. 2016;91(2).

569 27. Boeije H. Analysis in qualitative research. (Available in Dutch: Analyseren in kwalitatief onderzoek. Denken en doen). Den Haag: Boom Lemma uitgevers; 2014.

571 28. Pope C, Ziebland S, Mays N. Qualitative research in health care. Analysing qualitative data. BMJ. 2000;320:114-6.

573 29. MAXQDA, software for qualitative data analysis. Berling, Germany: VERBI Software - Consult - Socialforschung GmbH; 1989-2016.

575 30. Lewicki RJ, Bunker BB. Developing and maintaining trust in work relationships. In: Kramer R, Tyler T, editors. Trust in organizations. Thousand Oaks, CA: Sage; 1996. p. 114-39.

578 31. Tschannen-Moran M, Hoy WK. A multidisciplinary analysis of the nature, meaning, and measurement of trust. Rev Educ Res. 2000;70(4):547-93.

580 32. Gabarro JJ. The development of trust, influence, and expectations. In: Athos AG, Gabarro JJ, editors. Interpersonal behavior: Communication and understanding in relationships. Englewood Cliffs, NJ: Prentice Hall; 1978. p. 290-303.

583 33. McKnight DH, Cummings LL, Chervany NL. Initial trust formation in new organizational relationships. Acad Manage Rev. 1998;23:473-93.

585 34. Sutkin G, Wagner E, Harris I, Schiffer R. What makes a good clinical teacher in medicine? A review of literature. Academic medicine : journal of the Association of American Medical Colleges. 2008;83:452-66.

For peer review only - http://bmjopen.bmj.com/site/about/guidelines.xhtml

588 35. Tschannen-Moran M. Trust and collaboration in urban elementary schools. Columbus: Ohio State University; 1998.

590 36. Hirsh DA, Holmboe ES, ten Cate O. Time to trust: longitudinal integrated clerkships and entrustable professional activities. Academic medicine : journal of the Association of American Medical Colleges. 2014;89(2):201-4.

593 37. Bates J, Konkin J, Suddards C, Dobson S, Pratt D. Student perceptions of assessment and feedback in longitudinal integrated clekships. Medical Education. 2013;47:362-74.

595 38. Mazotti L, O'Brien B, Tong L, Hauer KE. Perceptions of evaluation in longitudinal versus traditional clerkships. Med Educ. 2011;45:464-70.

597

For peer review only

For peer review only - http://bmjopen.bmj.com/site/about/guidelines.xhtml

Tables

Table 1: topics used to facilitate focus group discussions	
**Opening question**	
• What do you need to trust someone in the context of training? What factors promote or undermine trust?	
**Key questions**	
**Trainers**	**Trainees**
• How does trust in a trainee evolve?	• How do you recognize your trainer trusting you?
• What factors are involved in the development of trust?	• How did this trust relationship evolve?
• How does the level of trust you show a trainee influence their work and learning in daily clinical practice?	• In what ways did you foster the development of trust?
• Is a trust relationship transferable to different individuals or clinical situations?	• How does the level of trust shown to you by your trainer influence your work and learning in daily clinical practice?
	• What factors would enable you to trust your trainer?
	• How does the level of trust you show your trainer influence your work and

For peer review only

	learning in daily clinical practice?

Table 2: Participant characteristics (Amsterdam, 2017)

		Trainers	Trainees
**Age in years (median (range))**		50.0 (39.0-62.0)	29.0 (27.0-38.0)
**Gender**			
	Female (%)	40.0%	82.4%
**Working experience in years**			
	As GP trainer (median (range))	8.0 (3.0-22.0)	-
	Before start GP training programme (median (range))	-	1.6 (0.0-4.5)

For peer review only

For peer review only - http://bmjopen.bmj.com/site/about/guidelines.xhtml

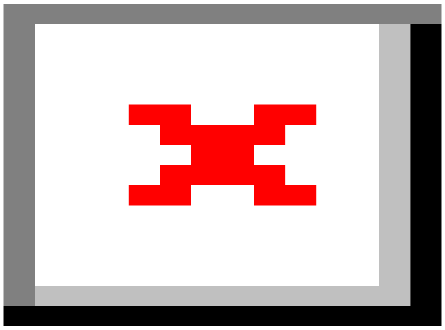

Figure 1: Model for the development of a mutual trust relationship

126x81mm (300 x 300 DPI)

BMJ Open

Original research: Insight in the development of the mutual trust relationship between trainers and trainees in a workplace-based post-graduate medical training programme: a focus group study among trainers and trainees of the Dutch General Practice training programme.

Journal:	*BMJ Open*
Manuscript ID	bmjopen-2019-036593.R1
Article Type:	Original research
Date Submitted by the Author:	16-Mar-2020
Complete List of Authors:	Bonnie, Linda; Amsterdam UMC - Locatie AMC, General Practice<br>Visser, Mechteld; Amsterdam UMC - Locatie AMC, Department of General Practice/GP Specialty Training<br>Kramer, Anneke ; Leiden University<br>van Dijk, Nynke; Amsterdam UMC - Locatie AMC
**Primary Subject Heading**:	Medical education and training
Secondary Subject Heading:	General practice / Family practice, Qualitative research
Keywords:	MEDICAL EDUCATION & TRAINING, QUALITATIVE RESEARCH, PRIMARY CARE

SCHOLARONE™
Manuscripts

I, the Submitting Author has the right to grant and does grant on behalf of all authors of the Work (as defined in the below author licence), an exclusive licence and/or a non-exclusive licence for contributions from authors who are: i) UK Crown employees; ii) where BMJ has agreed a CC-BY licence shall apply, and/or iii) in accordance with the terms applicable for US Federal Government officers or employees acting as part of their official duties; on a worldwide, perpetual, irrevocable, royalty-free basis to BMJ Publishing Group Ltd ("BMJ") its licensees and where the relevant Journal is co-owned by BMJ to the co-owners of the Journal, to publish the Work in this journal and any other BMJ products and to exploit all rights, as set out in our licence.

The Submitting Author accepts and understands that any supply made under these terms is made by BMJ to the Submitting Author unless you are acting as an employee on behalf of your employer or a postgraduate student of an affiliated institution which is paying any applicable article publishing charge ("APC") for Open Access articles. Where the Submitting Author wishes to make the Work available on an Open Access basis (and intends to pay the relevant APC), the terms of reuse of such Open Access shall be governed by a Creative Commons licence – details of these licences and which Creative Commons licence will apply to this Work are set out in our licence referred to above.

Other than as permitted in any relevant BMJ Author's Self Archiving Policies, I confirm this Work has not been accepted for publication elsewhere, is not being considered for publication elsewhere and does not duplicate material already published. I confirm all authors consent to publication of this Work and authorise the granting of this licence.

For peer review only

Original research: Insight in the development of the mutual trust relationship between trainers and trainees in a workplace-based post-graduate medical training programme: a focus group study among trainers and trainees of the Dutch General Practice training programme.

Authors

Linda H.A. Bonnie[a] (Msc MD), Mechteld R.M. Visser[a] (PhD), Anneke W.M. Kramer[b] (Professor, MD, PhD), Nynke van Dijk[a] (Professor, MD, PhD)

Affiliations

[a] Amsterdam UMC, Academic Medical Center; Department of General Practice; Amsterdam Public Health Research Institute; Amsterdam, The Netherlands

[b] Leiden University Medical Center; Department of Public Health and Primary Care; Leiden, The Netherlands

Corresponding author

Linda H.A. Bonnie, MsC MD

ORCID iD: 0000-0002-5581-8761

Institution: Amsterdam UMC, University of Amsterdam

Department: Department of General Practice, Amsterdam Public Health research institute

Adress: Meibergdreef 9, 1105 AZ Amsterdam, The Netherlands

Telephone number: 0031-20-5667165

For peer review only

For peer review only - http://bmjopen.bmj.com/site/about/guidelines.xhtml

E-mail address: l.h.bonnie@amsterdamumc.nl

For peer review only

For peer review only - http://bmjopen.bmj.com/site/about/guidelines.xhtml

Abstract

Objectives

Trust plays an important role in workplace-based postgraduate medical education (PGME) programmes. Trainers must trust their trainees for granting them greater independence. Trainees must trust their trainer for a safe learning environment. As trainers' and trainees' trust in each other plays an important role in trainee learning and development, the authors aimed to explore the development of the mutual trust relationship between trainers and trainees.

Setting

This study was performed in a General Practice training department in the Netherlands.

Participants

All trainers and trainees of the General Practice training department were invited to participate. Fifteen trainers and 34 trainees, voluntarily participated in focus group discussions.

Outcome measures

The authors aimed to gain insight in the factors involved in the development of the mutual trust relationship between trainers and trainees, in order to be able to create a model for the development of a mutual trust relationship between trainers and trainees.

Results

For peer review only

For peer review only - http://bmjopen.bmj.com/site/about/guidelines.xhtml

In the first stage of trust development, trainers and trainees develop basic trust in each other. Basic trust forms the foundation of the trust relationship. In the second stage, trainers develop trust in trainees taking into account trainees' working and learning performance, and the context in which the work is performed. Trainees trust their trainer based on the trainer's availability and accessibility and the personal relationship between the trainee and their trainer. Trainee self-confidence modifies the development of a trust relationship.

Conclusion

The development of a mutual trust relationship between trainers and trainees is a complex process that involves various stages, goals, factors, and interactive aspects. As the mutual trust relationship influences the learning environment for trainees, greater emphasis on the mutual trust relationship may improve learning outcomes. Further research may explore the effect of long-term and short-term educational relationships on the trust relationship between trainers and trainees.

For peer review only

For peer review only - http://bmjopen.bmj.com/site/about/guidelines.xhtml

Article Summary

Strengths and limitations of this study

- We included a relatively large amount of trainers and trainees in various stages of the training programme, which provided us with many different experiences and viewpoints.

- This study is a single-centre study, which may cause results to be not directly applicable in other PGME programs.

- Trainers included in the study were only involved in long educational relationships, so their vision on the influence of the duration of the educational relationship on mutual trust development could not be explored.

For peer review only - http://bmjopen.bmj.com/site/about/guidelines.xhtml

Funding

This publication was written as a part of the project "The use of Entrustable Professional Activities in Assessment in General Practice Specialty Training" (project number 839130004), that has received fundings from the "Netherlands Organisation for Health Research and Development" (ZonMW).

Competing interests

There are no competing interests for any author.

For peer review only

For peer review only - http://bmjopen.bmj.com/site/about/guidelines.xhtml

Introduction

Trust (*noun*): "Firm belief in the reliability, truth, or ability of someone or something".(1)

Entrust (*verb*): "To put one's trust in a person, with regard to a particular task or responsibility."(2)

Trust is a complex feeling, not easy to summarize in a simple definition. The amount of trust someone has in another person is highly dependent on personal factors, such as thoughts and motivations, as well as on the context in which the trust has to be awarded.(3-5) Additionally, trust is not an established fact, as it can be gradual and variable.(5)

Trust is fundamental to workplace-based learning in post graduate medical education (PGME) programmes.(3) In workplace-based PGME programmes, trainees learn to work independently when providing patient care, with the goal of becoming independently functioning medical specialists.(6, 7) In the process of learning to work independently, trainees are supervised by trainers, who are educators for trainees, next to their work as patient care providers.(8) In the process of supervision, trainers must balance the freedom they give their trainees in independently providing patient care, against patient safety. Trainers thus have to trust their trainee to provide good quality of care for their patients.(3, 9, 10) In this process of trust development, trainers take into account factors that are related to themselves as a trainer and as a patient care provider, the trainee, the trainer-trainee relationship, the context in which the work is performed and the nature of the task in question.(3, 10-15) Once trainers trust their trainee, they can gradually entrust them with performing patient care independently.(3)

For peer review only

Trust is not just important in terms of patient-safety, it is also a prerequisite for successful trainee learning. Being trusted by a trainer allows trainees to increasingly learn to work independently in the workplace and thereby develop themselves towards becoming independently functioning medical specialists.(3) Additionally, trainers entrusting trainees to perform tasks independently can boost motivation for learning and working.(3, 11) Also, for trainees being able to learn from experiences in the workplace, to perform their work properly, and being able to receive feedback, a safe learning environment is required. Trusting their trainer is essential for trainees to experience a safe learning environment.(16-20) However, factors involved in the process of trainees developing trust in their trainer still are poorly understood.

As both trainer's trust in their trainee(3), as well as trainee's trust in their trainer(16-20), play an important role in trainee learning and development, understanding the mutual trust relationship might help to optimize the learning outcomes for trainees.(16, 17) As factors involved in the process of trust development of trainees in their trainer still remain poorly understood, and little information is available about how the trust development processes of both trainers and trainees may influence each other, we aim to gain insight in the development of the mutual trust relationship between trainers and trainees in a workplace-based PGME-programme.

For peer review only

For peer review only - http://bmjopen.bmj.com/site/about/guidelines.xhtml

Methods

Context

This focus group study was conducted among the trainers and trainees of a Dutch GP-training programme. Dutch GPs are a pivotal element of the Dutch health-care system. In the event of a medical problem (life-threatening situations excluded), all Dutch inhabitants first visit their GP for advice and treatment. If necessary the GP refers to a medical specialist, for further evaluation.(21, 22) To prepare trainee GPs for this broad field of work, the Dutch GP-training program involves a significant amount of workplace-based learning. The first and third year of the training programme are spent working in general practice. During this period, trainees are supervised by one or two specially trained GPs, who coach, teach and assess the trainee. The second year of their training programme is dedicated to 3-6 month traineeships in the areas of clinical or emergency medicine, care for the elderly and psychiatry. The trainers in these internships are medical specialists who have been trained to supervise trainee GPs.(23, 24)

Study design

As we aimed to explore the process of development of the mutual trust relationship between trainers and trainees in a workplace-based PGME-program, we adopted a qualitative approach. As leading conceptual framework we chose to adopt a risk-based view of trust (Das and Teng, 2004)(25), as we assume that trust development is an ongoing process. We also assume that developing trust will have certain mutual aspects, because both parties involved in the trust development process have to take a risk in order to be open to each other's actions, and be able to develop trust in each other.(25) We chose to use focus group discussion with trainers and trainees for data collection, which we believed would provide insight in

For peer review only - http://bmjopen.bmj.com/site/about/guidelines.xhtml

participants' experiences and views regarding the development of trust. Individual differences in experience and views could be shared and explored in interactive group discussions. (26, 27)

For the analysis of the data, we used thematic analysis, as this is a method for "identifying, analysing, and reporting patterns within data".(28) With the thematic analysis we aimed to gain insight in the process of development of the mutual trust relationship between trainers and trainees. Although an extensive amount of literature is available about trainers developing trust in their trainees(3, 10-15), we chose to apply an inductive approach for the coding and analysis, as we hoped to identify factors involved in the development of the mutual trust relationship between trainers and trainees that currently are poorly understood an not yet described in the literature.(28, 29)

Participants

We conducted this study among GP trainers and trainee GPs from the Amsterdam UMC – location AMC GP training institute. We selected homogeneous groups, i.e. separate groups for trainers and trainees, as we expected that discussion of some aspects of trust could place participants in a vulnerable position, and we hoped that eliminating hierarchy in the focus groups would promote free and open discussions.(26)

All trainers and trainees from the Amsterdam UMC – location AMC GP training institute were informed about the purpose of the study, in person and by email, by the principle researcher (LB). Focus group meetings for trainers were held during the faculty development programme. Trainers could voluntary sign up for participation in the focus group discussions, after having received information about the study. Trainees participated in focus groups

For peer review only

For peer review only - http://bmjopen.bmj.com/site/about/guidelines.xhtml

during their weekly educational day, they could voluntarily sign up for participation in the focus group discussions after information about the study was provided to them.

Data collection

The data used in this study was obtained from a larger study into the implementation of Entrustable Professional Activities (EPAs) in the GP-training programme. The practical aspects of the use of EPAs in the GP-training programme are presented elsewhere.(30) Between November 2016 and March 2017, there were two trainer focus group discussions, and four trainee focus group discussions. Each focus group consisted of six to eleven participants, and lasted for 45-70 minutes. Demographic data (age, gender and length of career to date) on the participants was collected by means of a questionnaire.

Each focus group discussion was led by a skilled moderator (MV or NvD), while an assistant-moderator (LB or a research assistant) took notes.

The sessions started with a presentation on the purpose of the study, during which participants had the opportunity to ask questions. As a first step, we asked participants to explore their own definition of trust, and we encouraged participants to use their own definition of trust during the focus group discussion. In the next steps, an exploratory question initiated the discussion on trust between trainer and trainee, after which key questions were used to maintain the momentum. The topics covered (Table 1) were drawn from the literature on trust in workplace-based medical education.(3, 12, 14, 31) Data collection and data analysis were performed iteratively. The results of the data analysis were used to guide subsequent data collection.(26) The outcomes of the focus group discussions of both trainers and trainees were used to modify the topic list used in following focus group discussions with both trainers and

For peer review only - http://bmjopen.bmj.com/site/about/guidelines.xhtml

trainees, making it possible to explore trainers' and trainees' views and practical experience of trust in broader detail.

**Table 1: topics used to facilitate focus group discussions**	
**Opening question**	
• What do you need to trust someone in the context of training? What factors promote or undermine trust?	
**Key questions**	
**Trainers**	**Trainees**
• How does trust in a trainee evolve?	• How do you recognize your trainer trusting you?
• What factors are involved in the development of trust?	• How did this trust relationship evolve?
• How does the level of trust you show a trainee influence their work and learning in daily clinical practice?	• In what ways did you foster the development of trust?
• Is a trust relationship transferable to different individuals or clinical situations?	• How does the level of trust shown to you by your trainer influence your work and learning in daily clinical practice?
	• What factors would enable you to

For peer review only

For peer review only - http://bmjopen.bmj.com/site/about/guidelines.xhtml

	trust your trainer?
	• How does the level of trust you show your trainer influence your work and learning in daily clinical practice?

After each meeting, the moderator and assistant-moderator conducted a debriefing to discuss the course and the details of the discussion. The debriefing notes formed part of the dataset.

Data analysis

Audio recordings of the discussions were transcribed verbatim and anonymized. In the first step, LB (who is a GP-trainee) performed an inductive coding process in four focus group discussions. The first step in the coding process resulted in a code book with themes that emerged from the focus group discussions. The details of this code book were discussed at length with NvD (who is a MD and an educationalist), to ensure coding reliability. In the second step, LB and NvD performed an axial coding process involving the creation of overarching categories in the code book. In the third step, LB encoded the remaining two focus groups, using the code book derived from the axial coding process. As no new themes emerged from the last two focus groups, we assumed that data saturation had been reached. Once again, the code book was discussed in detail with NvD. The fourth step in the process of data analysis involved selective coding of the data. NvD, MV (who is a psychologist) and LB used the axial-coded code book to identify any themes that were relevant to the research question and to identify any relationships between the relevant themes. This process was supported by the use of memos and diagrams.(28, 32, 33) In the case of uncertainties or disagreement between the researchers during the coding process, the verbatim transcripts and audio-recorded data were consulted.

For peer review only - http://bmjopen.bmj.com/site/about/guidelines.xhtml

We used MAXQDA 2018 (VERBI Software (2017), Berlin, Germany) for data analysis.

Ethical approval

This study was approved by the Ethical Review Board of the Netherlands Association for Medical Education (ERB-NVMO, file-number 664). The participants were fully informed about the purpose of the study, participation was entirely voluntary, and individuals were able to withdraw at any time without having to give a reason. Prior to the focus group discussions, participants signed an informed consent form. All data was collected, stored and processed anonymously. No rewards were offered for participation in the study.

Patient and public involvement

There were no patients involved in this study.

For peer review only

Results

Fifteen GP trainers and 34 trainee GPs participated in the study. Participant characteristics are displayed in Table 2. Of the participating trainees, 82.4% was female. Overall, the atmosphere during the focus groups was pleasant. Participants felt safe to share their experiences, even though some experiences led to emotional reactions.

Table 2: Participant characteristics (Amsterdam, 2017)

		Trainers	Trainees
**Age in years (median (range))**		50.0 (39.0-62.0)	29.0 (27.0-38.0)
**Gender**			
	Female (%)	40.0%	82.4%
**Working experience in years**			
	As GP trainer (median (range))	8.0 (3.0-22.0)	-
	Before start GP training programme (median (range))	-	1.6 (0.0-4.5)

Based on our data we created a model that is based on the elements of the relationship between trainers and trainees that contribute to the development of a feeling of trust in trainers and trainees. (Figure 1). The development of trust involves multiple factors and stages, and also shows some interactive aspects. Trust development starts with the first impression, based on which both trainers and trainees develop a level of basic trust in one another. Further development of the trust relationship between trainers and trainees during the course of the

For peer review only - http://bmjopen.bmj.com/site/about/guidelines.xhtml

training programme is related to the process of training the trainee. Trainers and trainees adopt different approaches to building trust and have different goals. Trainers base the trust in their trainee on the performance of the trainee in working and learning in daily clinical practice, while trainees focus on the personal relationship with their trainer. Trainee self-confidence is a major factor affecting the development of the trust relationship in both directions. Below we further describe the components of the development of trust.

Stage 1: Basic trust

Based on their first impressions, trainers and trainees each start to develop basic trust in the other. Basic trust is not typically related to the training of the trainee, but entails a more generic kind of trust in other people. Basic trust forms the foundation of the trust relationship between trainers and trainees. The process of developing basic trust seems difficult to put into words. Both trainers and trainees refer to it as a "feeling", or "click" that they experience with the other person. They mention that the way of communicating with each other and a relaxed atmosphere during the first encounter play an important role in this process. Additionally, matching personality traits and a feeling of mutual understanding, also support the trust development process in this first short period.

Quote (trainee GP, 2nd year): "The development of trust starts with a good first impression. But it's not good if there is no 'click'."

Both trainers and trainees indicate that first impressions have a strong influence on their trust in each other. Once established, the trust relationship tends to be difficult to adjust, also when factors occur that might influence the degree of trust in the other person.

For peer review only - http://bmjopen.bmj.com/site/about/guidelines.xhtml

Quote (GP trainer): "There is very little scope for modifying a first impression. Your impression of the trainee turns out to be virtually immutable."

Stage 2: Trust development related to the training of the trainee

Once trainers and trainees start working together in daily clinical practice, the focus of the trust relationship shifts towards the process of training the trainee, based on experiences during working and learning in daily clinical practice. Trainers and trainees have different approaches to and different goals for building a trust relationship, but joint conditions for trust development are also present.

The development of a trainer's trust in their trainee

Trainers have to develop trust in their trainee in order to be able to entrust them to see patients independently. This trust is based on factors such as the trainee's performance in working and learning, context-dependent factors, and the degree of respect trainees show to patients, trainers and practice staff.

Trainee performance in working and learning is reflected in the trainee's transparency, their openness about how they perform their work, and their ability to learn from previous feedback. Trainers tend to focus on how trainees communicate about their positive and negative (learning) experiences in the workplace, on how they deal with trainer feedback, whether they know their own limitations, and ask for help at the right moments.

For peer review only - http://bmjopen.bmj.com/site/about/guidelines.xhtml

Quote (GP trainer): " Trust is also based on the trainee's openness about discussing past cases with me. If all I hear from them are success stories, while my assistant or patients give me conflicting reports, that gets me thinking: 'Wait a moment…'"

Quote (GP trainer): "We do not blindly trust trainees, believing them to be capable of dealing with any and all situations. We trust them because we know that, when they find themselves in situations of which they have no previous experience, they will be able to deal with them. They are aware of their own limitations, and they know when to ask for help."

Context-dependent factors also influence whether trainers place sufficient trust in their trainees to entrust them with care for specific patients. These include the nature of the patient's problem, and (special) situations in which the patient is presented. Trust is put to the test in emergency consultations, during house calls or during shifts at the Out-of-Hours GP Centre, when there is a greater risk of serious illness.

Quote (GP trainer): "Emergency care is typically a topic in which, as a trainer, I need to trust my trainee."

Quote (GP trainer): "In certain areas I think trainees can't do any harm, so I let them try to work independently."

<u>The development of a trainee's trust in their trainer</u>

Trainees have to trust their trainer in order to experience a safe learning environment. This trust is based on the trainer's availability and accessibility, and on the trainer's ability to create an environment in which the trainee feels that the trainer is willing to

For peer review only

listen to them and help them. If trainers are accessible, wherever and whenever required, this gives trainees a feeling of security, enhancing their trust in their trainer.

Quote (trainee GP, 2nd year): "I think it also has to do with accessibility. My GP trainer is very accessible, which increases my trust in him considerably."

Also, if trainers show a personal interest in their trainee, this creates a feeling of connectedness which, in turn, promotes the development of trust. Trainees find it more easy to be open about their performance and learning in daily clinical practice when they feel safe with, and connected to their trainer, especially in the case of problems and negative experiences.

Quote (trainee GP, 2nd year): "I did not have a good relationship with my trainer, there was no real click at the personal level, which made it harder for us to work together. It also made it harder for me to show my vulnerabilities…"

The trainees noticed that, during short, non-GP, rotations (three to six months), where several different trainers are involved in the training of the trainee, their personal relationship with their trainer was less intense than during year-long rotations in the GP-practice, with a maximum of two trainers. During short rotations, and in the case of multiple (more than two) trainers, trainees find it harder to develop a personal connection with, and trust in their trainers.

Quote (trainee GP, 2nd year): "In a whole year in training, you encounter all sorts of problems. It's good to discuss those problems with your trainer. If the individual

For peer review only - http://bmjopen.bmj.com/site/about/guidelines.xhtml

relationship were less intense, then I would not discuss my personal problems. On short rotations I notice that there is much less of a personal connection."

Joint conditions for trust development

Integrity - Both trainers and trainees build trust based on the integrity of the other person. Trainers and trainees assess each other's integrity in terms of consistent behaviour. They also compare each other's actions with their own work in daily practice and with their own moral standards. Consistent behaviour, and converging working styles and moral standards tend to boost their trust in each other.

Quote (GP trainer): "Sometimes, I don't trust a given trainee, but I don't know why. Usually, it has something to do with their Professional role. Sometimes I think, 'I would not approach someone in that way, I do not want my patients to be treated like that.' Yet, the trainee has done nothing wrong in terms of their Medical Expert role."
Quote (trainee GP, 2nd year): "I think, in fact, that it's easier to trust someone whose approach to work matches your own."

Trainee self-confidence - Trainee self-confidence is a major factor in the development of trust between trainers and trainees. If trainers entrust trainees to work independently in daily practice, this will improve trainee self-confidence. The effect is enhanced if trainers view trainees as equals and are willing to share their daily responsibilities with them.

If trainers and trainees have the same expectations of the trainee's performance, this boosts trainee self-confidence. However, if a trainee's degree of self-confidence

For peer review only - http://bmjopen.bmj.com/site/about/guidelines.xhtml

deviates from their trainer's expectations this can impair the trainer's trust, as trainers feel that too much or too little self-confidence could impact patient safety. Such mismatches between the trainer's expectations and the trainee's actual level of self-confidence can also impact the trainee's trust in their trainer, as in those cases trainees feel their trainer is not giving them sufficient support at the level where trainees feel they need it. As trainees are experiencing a lack of support from their trainer, this process will also negatively influence the self-confidence of the trainee.

Quote (GP trainer): "Some trainees tend to overestimate their abilities, and your immediate reaction is that you don't want to entrust your patients to them."
Quote (trainee GP, 1st year): "I get very uncomfortable if my trainer shows me a great deal of trust, because I wonder if I really deserve it."

For peer review only

For peer review only - http://bmjopen.bmj.com/site/about/guidelines.xhtml

Discussion

This study indicates that the process of mutual trust development between trainers and trainees in a workplace-based PGME-programme involves various stages, factors and interactive aspects. The process of mutual trust development starts at the first encounter, with the first impression forming basic trust. Once trainers and trainees start working together in daily clinical practice, the focus of the trust development process shifts towards working and learning in the workplace. In this phase, trainers develop trust in their trainees in order to be able to entrust them with patient care. Trainees need to trust their trainer in order to experience a safe learning environment. Next to trainer- and trainee-related factors, joint conditions for mutual trust development, like integrity and trainee self-confidence, are also involved in the mutual trust development process.

The process of developing a trust relationship between two people knows various stages. In the first stage, the involved persons have to get to know each other. Once they have become familiar with each other, the trust development process will increasingly be based on the knowledge about the other person and about the relationship that they have developed.(34) Tschannen-Moran and Hoy(35) assume that learners follow four stages in developing trust in their teachers: the initial impression phase, the exploration phase, the limit testing phase, and the stability phase.(35, 36) The findings of our study mainly reflect the initial impression stage (basic trust development) and the exploration and limit-testing stage (trust development focusing on working and learning in daily practice). In our study, the stage of development of basic trust (initial impression stage), seems to have a strong influence on further trust development process. This is in line with other studies saying that the amount of trust

For peer review only - http://bmjopen.bmj.com/site/about/guidelines.xhtml

developed in the initial stage influences the likelihood to see factors and situations that further influence trust.(37)

In the second stage of trust development, when the trust development process focusses on working and learning in daily practice, trainers and trainees have different styles in trusting each other, and different goals for trusting each other. The factors influencing the trust of trainers in their trainees were the same ones identified by Hauer, et al. (14), and Sagasser, et al. (15) in their studies into this process. Trainees in the second stage of trust development develop trust in their trainer based on the availability and accessibility of the trainer for the trainee, and on their personal relationship. This is in line with factors that Sutkin et al. identified as students' criteria for good clinical teachers(38), and with earlier findings that a caring attitude towards learners may support trust development from trainees in their trainer.(35, 39) This might explain why trainees, more than trainers, value the personal relationship when developing trust in their trainer.

The trainees noticed that the trust relationship with their trainer was stronger during long rotations (a minimum of one year) than it was during short rotations (usually three to six months), especially due to the fact that they were able to develop a more intense personal relationship with their trainer. Hirsh et al. found that this effect may be due to the more effective distribution of time during longitudinal rotations.(40) A more intense personal relationship during long rotations encourages trainees to discuss their performance with their trainer more often, leading to greater shared responsibility in identifying approaches to deal with any weaknesses in the trainee's performance. It also helped trainees to take a more constructive view of their trainer's feedback and to provide better patient care.(41) Longitudinal rotations may therefore not only lead to an improved learning environment with

For peer review only - http://bmjopen.bmj.com/site/about/guidelines.xhtml

more learning opportunities for the trainee, it might also help to improve patient care. Other explanations for the difference between long and short rotations could be the differences in culture between working in hospitals and working in GP-practices, as well as power-differences and ethnocentric mismatches between GP-trainees and their trainers in hospitals.

Strengths and limitations

For this study we were able to include a relatively large amount of trainers and trainees, in various stages of the training programme, which provided us with insights in many different experiences and viewpoints about the trust relationship between trainers and trainees.

Trust is a feeling, that might have a different definition for every single person. (4, 5, 42)This might cause challenges in investigating trust development. Although the feeling of trust might be very personal, participants in this study indicate the same factors involved in trust as reflected in available literature.(14, 15, 34-39) We therefore think that the results of this study are representative for the trust development process between trainers and trainees.

Of the participating trainees, 82.4% was female. Since 77% of trainee GPs in the Netherlands are female(43), females were slightly overrepresented in our study. We do not think that the results of our study are influenced by this variation and feel that the results are representative for Dutch trainee GPs.

This study is a single-centre study, focussing on the development of a trust relationship in the GP training programme. Therefore, the results might not be directly applicable in other PGME programs. We were however able to identify factors that might influence the trust development process in (non-GP) short rotations or rotations with multiple trainers. We

For peer review only - http://bmjopen.bmj.com/site/about/guidelines.xhtml

477 therefore think that aspects of our study may help various kinds of PGME programmes to
478 further optimize the learning environment for their trainees.
479
480 **Future research**
481 The trainees provided us with important insights concerning the effect of an educational
482 relationship's duration on the trust relationship between trainers and trainees. However, we do
483 not know which factors involved in short and long educational relationships influence the
484 trust relationship between trainers and trainees. Additionally, in this study we did not explore
485 the vision of trainers involved in short rotations. The distinction between long-term and short-
486 term educational relationships and their effect on the trust relationships between trainers and
487 trainees were therefore not fully explored and merit further study.
488
489 **Implications for practice**
490 During the first impression, the foundation is made for the trust relationship between trainers
491 and trainees. By introducing trainers and trainees to each other before the start of a training
492 period, a solid foundation for the trust relationship can be established. When trainers and
493 trainees are aware of the halo-effect of trust and of the differences in their needs concerning
494 the trust relationship, the trust relationship, and thereby learning in practice can be enhanced.
495
496 Furthermore, a long duration of a training relationship positively influences the trust
497 relationship between trainers and trainees when compared to short-term training relationships,
498 which might improve trainee performance and patient care.(41, 44) When long-term training
499 relationships are difficult to realize, the findings of this study might help trainers in short-term
500 training relationships to improve the trust relationship between trainers and trainees, which
501 could also improve the learning environment for trainees and patient care.

For peer review only

For peer review only - http://bmjopen.bmj.com/site/about/guidelines.xhtml

For peer review only

For peer review only - http://bmjopen.bmj.com/site/about/guidelines.xhtml

504 # Figures

505 **Figure 1:** Model for the mutual trust relationship between trainers and trainees.

506

For peer review only

For peer review only - http://bmjopen.bmj.com/site/about/guidelines.xhtml

Acknowledgements

The authors wish to thank the participating trainers and trainees for their willingness to contribute to the study.

Author's contribution

Linda H.A. Bonnie: Substantial contribution to the conception and design of the work, and to the acquisition, analysis and interpretation of data. Substantial contribution in drafting the work. Provides final approval of the version to be published. Agrees to be accountable for all aspects of the work.

Mechteld R.M. Visser: Substantial contribution to the acquisition, analysis and interpretation of data. Substantial contribution in critically revising the work. Provides final approval of the version to be published. Agrees to be accountable for all aspects of the work.

Anna W.M. Kramer: Substantial contribution to the conception of the work and the interpretation of data. Substantial contribution in critically revising the work. Provides final approval of the version to be published. Agrees to be accountable for all aspects of the work.

Nynke van Dijk: Substantial contribution to the conception and design of the work, and to the acquisition, analysis and interpretation of data. Substantial contribution in drafting and critically revising the work. Provides final approval of the version to be published. Agrees to be accountable for all aspects of the work.

Data sharing

The data for this paper may be obtained from the authors upon request.

For peer review only - http://bmjopen.bmj.com/site/about/guidelines.xhtml

Word count

Abstract: 301

Main text: 4395

Number of figures: 1

Number of tables: 2

For peer review only

For peer review only - http://bmjopen.bmj.com/site/about/guidelines.xhtml

Literature

1. Oxford Dictionaries [Definition for "Trust".]. Available from: https://www.oed.com/view/Entry/207004?rskey=RegA4O&result=1#eid.
2. Oxford Dictionaries [Definition for "Entrust"]. Available from: https://www.oed.com/view/Entry/63012?redirectedFrom=entrust#eid.
3. Hauer KE, Ten Cate O, Boscardin C, Irby DM, Iobst W, O'Sullivan PS. Understanding trust as an essential element of trainee supervision and learning in the workplace. Adv Health Sci Educ Theory Pract. 2014;19(3):435-56.
4. Burke CS, Ims DE, Lazzara EH, Salas E. Trust in leadership: A multi-level review and integration. The Leadership Quarterly. 2007;18:606-32.
5. Dietz G, Den Hartog DN. Measuring trust inside organisations. Personnel Review. 2006;35:557-88.
6. Kennedy TJ, Regehr G, Baker GR, Lingard LA. Progressive independence in clinical training: a tradition worth defending? Academic medicine : journal of the Association of American Medical Colleges. 2005;80:S106-S11.
7. Dornan T, Boshuizen H, King N, Scherpbier A. Experience-based learning: a model linking the processes and outcomes of medical students' workplace learning. Med Educ. 2007;41(1):84-91.
8. Taylor EW, Tisdell EJ, Gusic ME. Teaching beliefs of medical educators: perspectives on clinical teaching in pediatrics. Medical teacher. 2007;29:371-6.
9. Carracio C, Englander R, Holmboe ES, Kogan JR. Driving care quality: Aligning trainee assessment and supervision through practical application of entrustable professional activities, competencies, and milestones. . Academic medicine : journal of the Association of American Medical Colleges. 2016;91:199-203.
10. ten Cate O, Scheele F. Viewpoint: competency-based postgraduate training: can we bridge the gap between theory and clinical practice? Academic medicine : journal of the Association of American Medical Colleges. 2007;82(6):542-7.
11. Sterkenburg A, Barach P, Kalkman C, Gielen M, ten Cate O. When do supervising physicians decide to entrust residents with unsupervised tasks? Academic medicine : journal of the Association of American Medical Colleges. 2010;85(9):1408-17.
12. Choo KJ, Arora VM, Barach P, Johnson JK, Farnan JM. How do supervising physicians decide to entrust residents with unsupervised tasks? A qualitative analysis. J Hosp Med. 2014;9(3):169-75.
13. Dijksterhuis MG, Voorhuis M, Teunissen PW, Schuwirth LW, ten Cate OT, Braat DD, et al. Assessment of competence and progressive independence in postgraduate clinical training. Med Educ. 2009;43(12):1156-65.
14. Hauer KE, Oza SK, Kogan JR, Stankiewicz CA, Stenfors-Hayes T, Cate OT, et al. How clinical supervisors develop trust in their trainees: a qualitative study. Med Educ. 2015;49(8):783-95.
15. Sagasser MH, Fluit CRMG, van Weel C, van der Vleuten CPM, Kramer AWM. How entrustment is informed by holistic judgements across time in a family medcne residency program: an ethnographic nonparticipant observational study. Academic medicine : journal of the Association of American Medical Colleges. Epub ahead of print.
16. Frandsen Gran S, A.M. B, Lindbaek M, Frich JC. General practicioners'and students'experiences with feedback during a six-week clerkship in general practice: a qualitative study. Scand J Prim Health Care. 2016;34(2):171-8.
17. Sklar DP. Trust is a two-way street. Academic medicine : journal of the Association of American Medical Colleges. 2016;91:155-8.
18. Carless D. Trust, distrust and their impact on assessment reform. Assessment & Evaluation in Higher Education. 2009;34(1):79-89.
19. Evans C. Making sense of assessment feedback in higher education. Rev Educ Res. 2013;83(1):70-120.

For peer review only - http://bmjopen.bmj.com/site/about/guidelines.xhtml

20. Epstein RM. Assessment in medical education. N Engl J Med. 2007;356(4):387-96.
21. Publiek Z. General Practice care (Available in Dutch: Huisartsenzorg) 2015 [March 2016]. Available from: https://www.zorgprismapubliek.nl/informatie-over/huisartsenzorg/huisartsenzorg/.
22. van Weel C. Primary health care and family medicine at the core of health care: challanges and priorities in how to further strengthen their potential. Front Med. 2014;1(37):1-5.
23. Huisartsopleiding Nederland. What does the training program look like? (Available in Dutch: Hoe ziet de opleiding eruit?) [09-12-2015]. Available from: http://uitdepraktijk.huisartsopleiding.nl/opleiding/hoe-ziet-de-opleiding-eruit.
24. College voor Huisartsgeneeskunde, Verpleeghuisgeneeskunde en medische zorg voor verstandelijk gehandicapten. Decree on general practice medicine regarding registration requirements for general practitioners. (Available in Dutch: Besluit huisartsgeneeskunde; Besluit van 21 augustus 2008 houdende opleidings, erkennings- en (her)registratie-eisen voor het specialisme huisartsgeneeskunde), (2015).
25. Das TK, B-S. T. The risk-based view of trust: a conceptual framework. Journal of Business and Psychology. 2004;19(1):85-116.
26. Stalmeijer RE, McNaughton N, Van Mook WN. Using focus groups in medical education research: AMEE Guide No. 91. Medical teacher. 2014;36(11):923-39.
27. Kitzinger J. Qualitative research. Introducing focus groups. BMJ. 1995;311(7000):299.
28. Braun V, Clarke V. Using thematic analysis in psychology. Qual Res Psychol. 2006;3(2):77-101.
29. Braun V, Clarke V. Thematic analysis. In: Cooper H, editor. APA handbook of research methods in psychology. 2. Research designs: American Psychological Association; 2012. p. 57-70.
30. Bonnie LHA, Visser MRM, Bont J, Kramer AWM, van Dijk N. Trainers' and Trainees' expectations of Entrustable Professional Activities (EPAs) in a Primary Care training programme. . Education for Primary Care. 2019;30(1):13-21.
31. ten Cate O, Hart D, Ankel F, Busari J, Englander R, Glasgow N, et al. Entrustment Decision Making in Clinical Training. Academic medicine : journal of the Association of American Medical Colleges. 2016;91(2).
32. Boeije H. Analysis in qualitative research. (Available in Dutch: Analyseren in kwalitatief onderzoek. Denken en doen). Den Haag: Boom Lemma uitgevers; 2014.
33. Pope C, Ziebland S, Mays N. Qualitative research in health care. Analysing qualitative data. BMJ. 2000;320:114-6.
34. Lewicki RJ, Bunker BB. Developing and maintaining trust in work relationships. In: Kramer R, Tyler T, editors. Trust in organizations. Thousand Oaks, CA: Sage; 1996. p. 114-39.
35. Tschannen-Moran M, Hoy WK. A multidisciplinary analysis of the nature, meaning, and measurement of trust. Rev Educ Res. 2000;70(4):547-93.
36. Gabarro JJ. The development of trust, influence, and expectations. In: Athos AG, Gabarro JJ, editors. Interpersonal behavior: Communication and understanding in relationships. Englewood Cliffs, NJ: Prentice Hall; 1978. p. 290-303.
37. McKnight DH, Cummings LL, Chervany NL. Initial trust formation in new organizational relationships. Acad Manage Rev. 1998;23:473-93.
38. Sutkin G, Wagner E, Harris I, Schiffer R. What makes a good clinical teacher in medicine? A review of literature. Academic medicine : journal of the Association of American Medical Colleges. 2008;83:452-66.
39. Tschannen-Moran M. Trust and collaboration in urban elementary schools. Columbus: Ohio State University; 1998.
40. Hirsh DA, Holmboe ES, ten Cate O. Time to trust: longitudinal integrated clerkships and entrustable professional activities. Academic medicine : journal of the Association of American Medical Colleges. 2014;89(2):201-4.
41. Bates J, Konkin J, Suddards C, Dobson S, Pratt D. Student perceptions of assessment and feedback in longitudinal integrated clekships. Medical Education. 2013;47:362-74.

For peer review only - http://bmjopen.bmj.com/site/about/guidelines.xhtml

42. Hauer KE, Soni K, Cornett P, Kohlwes J, Hollander H, Ranji SR, et al. Developing entrustable professional activities as the basis for assessment of competence in an internal medicine residency: a feasibility study. J Gen Intern Med. 2013;28(8):1110-4.
43. Versteeg S, Batenburg R. Figures from the registration of General Practitioners, Survey 2017. (Available in Dutch: Cijfers uit de registratie van huisartsen, Peiling 2017). Nivel; 2019.
44. Mazotti L, O'Brien B, Tong L, Hauer KE. Perceptions of evaluation in longitudinal versus traditional clerkships. Med Educ. 2011;45:464-70.

For peer review only

For peer review only - http://bmjopen.bmj.com/site/about/guidelines.xhtml

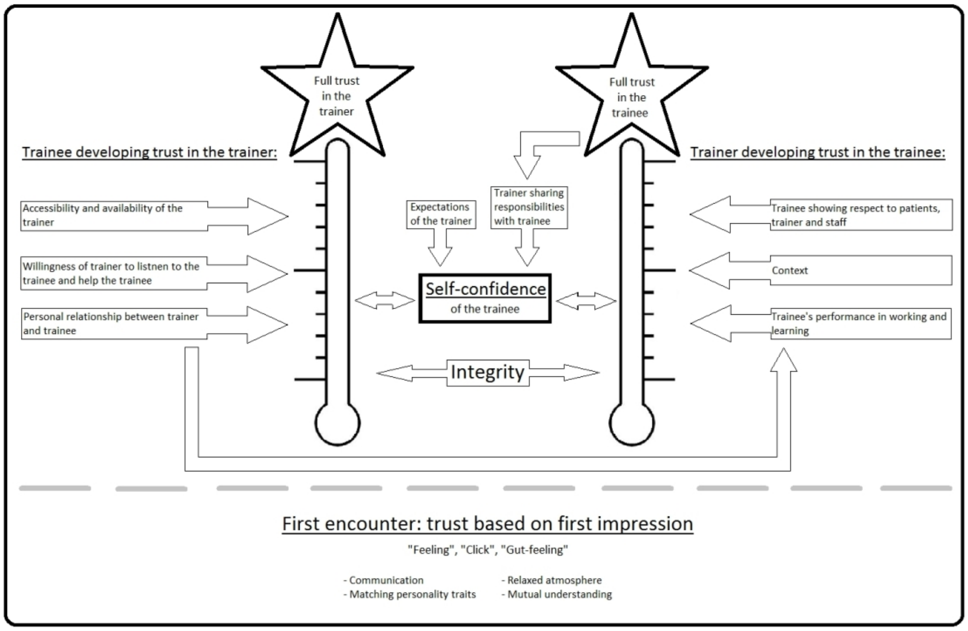

Figure 1, Model for the development of a mutual trust relationship.

126x81mm (300 x 300 DPI)

COREQ (COnsolidated criteria for REporting Qualitative research) Checklist

A checklist of items that should be included in reports of qualitative research. You must report the page number in your manuscript where you consider each of the items listed in this checklist. If you have not included this information, either revise your manuscript accordingly before submitting or note N/A.

Topic	Item No.	Guide Questions/Description	Reported on Page No.
**Domain 1: Research team and reflexivity**			
Personal characteristics			
Interviewer/facilitator	1	Which author/s conducted the interview or focus group?	11 line 195-19[illegible]
Credentials	2	What were the researcher's credentials? E.g. PhD, MD	1 line 8-9
Occupation	3	What was their occupation at the time of the study?	1 line 216-229
Gender	4	Was the researcher male or female?	n/a
Experience and training	5	What experience or training did the researcher have?	12 line 216-22[illegible]
Relationship with participants			
Relationship established	6	Was a relationship established prior to study commencement?	n/a
Participant knowledge of the interviewer	7	What did the participants know about the researcher? e.g. personal goals, reasons for doing the research	n/a
Interviewer characteristics	8	What characteristics were reported about the inter viewer/facilitator? e.g. Bias, assumptions, reasons and interests in the research topic	none
**Domain 2: Study design**			
Theoretical framework			
Methodological orientation and Theory	9	What methodological orientation was stated to underpin the study? e.g. grounded theory, discourse analysis, ethnography, phenomenology, content analysis	10 line 162-16[illegible]
Participant selection			
Sampling	10	How were participants selected? e.g. purposive, convenience, consecutive, snowball	10-11 line 172-[illegible]
Method of approach	11	How were participants approached? e.g. face-to-face, telephone, mail, email	10 line 172-18[illegible]
Sample size	12	How many participants were in the study?	15 line 246-24[illegible]
Non-participation	13	How many people refused to participate or dropped out? Reasons?	n/a
Setting			
Setting of data collection	14	Where was the data collected? e.g. home, clinic, workplace	10-11 line 172-[illegible]
Presence of non-participants	15	Was anyone else present besides the participants and researchers?	11 line 195-19[illegible]
Description of sample	16	What are the important characteristics of the sample? e.g. demographic data, date	15 line 246-24[illegible]
Data collection			
Interview guide	17	Were questions, prompts, guides provided by the authors? Was it pilot tested?	11-13 line 198-[illegible]
Repeat interviews	18	Were repeat inter views carried out? If yes, how many?	no
Audio/visual recording	19	Did the research use audio or visual recording to collect the data?	13 line 216
Field notes	20	Were field notes made during and/or after the inter view or focus group?	11 line 195-19[illegible]
Duration	21	What was the duration of the inter views or focus group?	11 line 190-19[illegible]
Data saturation	22	Was data saturation discussed?	123line 223-22[illegible]
Transcripts returned	23	Were transcripts returned to participants for comment and/or	no

For peer review only

For peer review only - http://bmjopen.bmj.com/site/about/guidelines.xhtml

Topic	Item No.	Guide Questions/Description	Reported on Page No.
		correction?	
**Domain 3: analysis and findings**			
Data analysis			
Number of data coders	24	How many data coders coded the data?	13 line 216-231
Description of the coding tree	25	Did authors provide a description of the coding tree?	no
Derivation of themes	26	Were themes identified in advance or derived from the data?	10 line 162-169
Software	27	What software, if applicable, was used to manage the data?	14 line 233
Participant checking	28	Did participants provide feedback on the findings?	no
Reporting			
Quotations presented	29	Were participant quotations presented to illustrate the themes/findings? Was each quotation identified? e.g. participant number	pp 16-21
Data and findings consistent	30	Was there consistency between the data presented and the findings?	pp 15-25
Clarity of major themes	31	Were major themes clearly presented in the findings?	pp 15-21
Clarity of minor themes	32	Is there a description of diverse cases or discussion of minor themes?	pp 15-25

Developed from: Tong A, Sainsbury P, Craig J. Consolidated criteria for reporting qualitative research (COREQ): a 32-item checklist for interviews and focus groups. *International Journal for Quality in Health Care*. 2007. Volume 19, Number 6: pp. 349 – 357

Once you have completed this checklist, please save a copy and upload it as part of your submission. DO NOT include this checklist as part of the main manuscript document. It must be uploaded as a separate file.

For peer review only

BMJ Open

Insight in the development of the mutual trust relationship between trainers and trainees in a workplace-based post-graduate medical training programme: a focus group study among trainers and trainees of the Dutch General Practice training programme.

Journal:	*BMJ Open*
Manuscript ID	bmjopen-2019-036593.R2
Article Type:	Original research
Date Submitted by the Author:	03-Apr-2020
Complete List of Authors:	Bonnie, Linda; Amsterdam UMC - Locatie AMC, General Practice<br>Visser, Mechteld; Amsterdam UMC - Locatie AMC, Department of General Practice/GP Specialty Training<br>Kramer, Anneke ; Leiden University<br>van Dijk, Nynke; Amsterdam UMC - Locatie AMC
**Primary Subject Heading**:	Medical education and training
Secondary Subject Heading:	General practice / Family practice, Qualitative research
Keywords:	MEDICAL EDUCATION & TRAINING, QUALITATIVE RESEARCH, PRIMARY CARE

SCHOLARONE™
Manuscripts

I, the Submitting Author has the right to grant and does grant on behalf of all authors of the Work (as defined in the below author licence), an exclusive licence and/or a non-exclusive licence for contributions from authors who are: i) UK Crown employees; ii) where BMJ has agreed a CC-BY licence shall apply, and/or iii) in accordance with the terms applicable for US Federal Government officers or employees acting as part of their official duties; on a worldwide, perpetual, irrevocable, royalty-free basis to BMJ Publishing Group Ltd ("BMJ") its licensees and where the relevant Journal is co-owned by BMJ to the co-owners of the Journal, to publish the Work in this journal and any other BMJ products and to exploit all rights, as set out in our licence.

The Submitting Author accepts and understands that any supply made under these terms is made by BMJ to the Submitting Author unless you are acting as an employee on behalf of your employer or a postgraduate student of an affiliated institution which is paying any applicable article publishing charge ("APC") for Open Access articles. Where the Submitting Author wishes to make the Work available on an Open Access basis (and intends to pay the relevant APC), the terms of reuse of such Open Access shall be governed by a Creative Commons licence – details of these licences and which Creative Commons licence will apply to this Work are set out in our licence referred to above.

Other than as permitted in any relevant BMJ Author's Self Archiving Policies, I confirm this Work has not been accepted for publication elsewhere, is not being considered for publication elsewhere and does not duplicate material already published. I confirm all authors consent to publication of this Work and authorise the granting of this licence.

For peer review only

Insight in the development of the mutual trust relationship between trainers and trainees in a workplace-based post-graduate medical training programme: a focus group study among trainers and trainees of the Dutch General Practice training programme.

Authors

Linda H.A. Bonnie[a] (Msc MD), Mechteld R.M. Visser[a] (PhD), Anneke W.M. Kramer[b] (Professor, MD, PhD), Nynke van Dijk[a] (Professor, MD, PhD)

Affiliations

[a] Amsterdam UMC, Academic Medical Center; Department of General Practice; Amsterdam Public Health Research Institute; Amsterdam, The Netherlands

[b] Leiden University Medical Center; Department of Public Health and Primary Care; Leiden, The Netherlands

Corresponding author

Linda H.A. Bonnie, MsC MD

ORCID iD: 0000-0002-5581-8761

Institution: Amsterdam UMC, University of Amsterdam

Department: Department of General Practice, Amsterdam Public Health research institute

Adress: Meibergdreef 9, 1105 AZ Amsterdam, The Netherlands

Telephone number: 0031-20-5667165

E-mail address: l.h.bonnie@amsterdamumc.nl

For peer review only - http://bmjopen.bmj.com/site/about/guidelines.xhtml

Abstract

Objectives

Trust plays an important role in workplace-based postgraduate medical education (PGME) programmes. Trainers must trust their trainees for granting them greater independence. Trainees must trust their trainer for a safe learning environment. As trainers' and trainees' trust in each other plays an important role in trainee learning and development, the authors aimed to explore the development of the mutual trust relationship between trainers and trainees.

Setting

This study was performed in a General Practice training department in the Netherlands.

Participants

All trainers and trainees of the General Practice training department were invited to participate. Fifteen trainers and 34 trainees, voluntarily participated in focus group discussions.

Outcome measures

The authors aimed to gain insight in the factors involved in the development of the mutual trust relationship between trainers and trainees, in order to be able to create a model for the development of a mutual trust relationship between trainers and trainees. The risk-based view of trust was adopted as leading conceptual framework.

Results

For peer review only - http://bmjopen.bmj.com/site/about/guidelines.xhtml

In the first stage of trust development, trainers and trainees develop basic trust in each other. Basic trust forms the foundation of the trust relationship. In the second stage, trainers develop trust in trainees taking into account trainees' working and learning performance, and the context in which the work is performed. Trainees trust their trainer based on the trainer's availability and accessibility and the personal relationship between the trainee and their trainer. Trainee self-confidence modifies the development of a trust relationship.

Conclusion

The development of a mutual trust relationship between trainers and trainees is a complex process that involves various stages, goals, factors, and interactive aspects. As the mutual trust relationship influences the learning environment for trainees, greater emphasis on the mutual trust relationship may improve learning outcomes. Further research may explore the effect of long-term and short-term educational relationships on the trust relationship between trainers and trainees.

For peer review only

For peer review only - http://bmjopen.bmj.com/site/about/guidelines.xhtml

Article Summary

- **Strengths and limitations of this study**We used the risk-based view of trust as the leading conceptual framework for this study.

- We included a relatively large amount of trainers and trainees in various stages of the training programme, which provided us with many different experiences and viewpoints.

- This study is a single-centre study, which may cause results to be not directly applicable in other PGME programs.

- Trainers included in the study were only involved in long educational relationships, so their vision on the influence of the duration of the educational relationship on mutual trust development could not be explored.

For peer review only

Funding

This publication was written as a part of the project “The use of Entrustable Professional Activities in Assessment in General Practice Specialty Training” (project number 839130004), that has received fundings from the “Netherlands Organisation for Health Research and Development” (ZonMW).

Competing interests

There are no competing interests for any author.

For peer review only

For peer review only - http://bmjopen.bmj.com/site/about/guidelines.xhtml

Introduction

Trust (*noun*): "Firm belief in the reliability, truth, or ability of someone or something".(1)

Entrust (*verb*): "To put one's trust in a person, with regard to a particular task or responsibility."(2)

Trust is a complex feeling, not easy to summarize in a simple definition. The amount of trust someone has in another person is highly dependent on personal factors, such as thoughts and motivations, as well as on the context in which the trust has to be awarded.(3-5) Additionally, trust is not an established fact, as it can be gradual and variable.(5)

Trust is fundamental to workplace-based learning in post graduate medical education (PGME) programmes.(3) In workplace-based PGME programmes, trainees learn to work independently when providing patient care, with the goal of becoming independently functioning medical specialists.(6, 7) In the process of learning to work independently, trainees are supervised by trainers, who are educators for trainees, next to their work as patient care providers.(8) In the process of supervision, trainers must balance the freedom they give their trainees in independently providing patient care, against patient safety. Trainers thus have to trust their trainee to provide good quality of care for their patients.(3, 9, 10) In this process of trust development, trainers take into account factors that are related to themselves as a trainer and as a patient care provider, the trainee, the trainer-trainee relationship, the context in which the work is performed and the nature of the task in question.(3, 10-15) Once trainers trust their trainee, they can gradually entrust them with performing patient care independently.(3)

For peer review only

For peer review only - http://bmjopen.bmj.com/site/about/guidelines.xhtml

Trust is not just important in terms of patient-safety, it is also a prerequisite for successful trainee learning. Being trusted by a trainer allows trainees to increasingly learn to work independently in the workplace and thereby develop themselves towards becoming independently functioning medical specialists.(3) Additionally, trainers entrusting trainees to perform tasks independently can boost motivation for learning and working.(3, 11) Also, for trainees being able to learn from experiences in the workplace, to perform their work properly, and being able to receive feedback, a safe learning environment is required. Trusting their trainer is essential for trainees to experience a safe learning environment.(16-20) However, factors involved in the process of trainees developing trust in their trainer still are poorly understood.

As both trainer's trust in their trainee(3), as well as trainee's trust in their trainer(16-20), play an important role in trainee learning and development, understanding the mutual trust relationship might help to optimize the learning outcomes for trainees.(16, 17) As factors involved in the process of trust development of trainees in their trainer still remain poorly understood, and little information is available about how the trust development processes of both trainers and trainees may influence each other, we aim to gain insight in the development of the mutual trust relationship between trainers and trainees in a workplace-based PGME-programme.

For peer review only - http://bmjopen.bmj.com/site/about/guidelines.xhtml

Methods

Context

This focus group study was conducted among the trainers and trainees of a Dutch GP-training programme. Dutch GPs are a pivotal element of the Dutch health-care system. In the event of a medical problem (life-threatening situations excluded), all Dutch inhabitants first visit their GP for advice and treatment. If necessary the GP refers to a medical specialist, for further evaluation.(21, 22) To prepare trainee GPs for this broad field of work, the Dutch GP-training program involves a significant amount of workplace-based learning. The first and third year of the training programme are spent working in general practice. During this period, trainees are supervised by one or two specially trained GPs, who coach, teach and assess the trainee. The second year of their training programme is dedicated to 3-6 month traineeships in the areas of clinical or emergency medicine, care for the elderly and psychiatry. The trainers in these internships are medical specialists who have been trained to supervise trainee GPs.(23, 24)

Study design

As we aimed to explore the process of development of the mutual trust relationship between trainers and trainees in a workplace-based PGME-program, we adopted a qualitative approach. As leading conceptual framework we chose to adopt a risk-based view of trust (Das and Teng, 2004).(25) By using this framework, we assume that trust and risk are theoretically each other's opposites. This means that when we trust someone, we experience a low risk in trusting the other person (perceived risk), and we are willing to accept that risk. On the other hand, when we do not trust someone, the perceived risk in trusting the other person is high, and we are not willing to accept that risk. The feeling of a certain amount of trust in someone

For peer review only - http://bmjopen.bmj.com/site/about/guidelines.xhtml

is called subjective trust, the perception of trust. This subjective trust is influenced by personal and situational factors, the so-called trust antecedents in the terminology of Das and Teng.(25) The interplay between subjective trust and trust antecedents leads to certain behaviour in taking risks and trusting someone, also known as behavioural trust.(25)

Within the framework of a risk-based view of trust, the consequences of behavioural trust may again inform and influence a person's subjective trust. Additionally, Das and Teng believe that trusting somebody, and therefore taking a risk in that person, can boost the development of a mutual trust relationship.(25) We use these assumptions as the basis of our study, as we aim to explore how the mutual trust relationship between trainers and trainees develops, and which factors are involved in this process.

We chose to use focus group discussion with trainers and trainees for data collection, which we believed would provide insight in participants' experiences and views regarding the development of trust. Individual differences in experience and views could be shared and explored in interactive group discussions. (26, 27)

For the analysis of the data, we used thematic analysis, as this is a method for "identifying, analysing, and reporting patterns within data".(28) With the thematic analysis we aimed to gain insight in the process of development of the mutual trust relationship between trainers and trainees. Although an extensive amount of literature is available about trainers developing trust in their trainees(3, 10-15), we chose to apply an inductive approach for the coding and analysis, as we hoped to identify factors involved in the development of the mutual trust relationship between trainers and trainees that currently are poorly understood an not yet described in the literature.(28, 29)

For peer review only - http://bmjopen.bmj.com/site/about/guidelines.xhtml

Participants

We conducted this study among GP trainers and trainee GPs from the Amsterdam UMC – location AMC GP training institute. We selected homogeneous groups, i.e. separate groups for trainers and trainees, as we expected that discussion of some aspects of trust could place participants in a vulnerable position, and we hoped that eliminating hierarchy in the focus groups would promote free and open discussions.(26)

All trainers and trainees from the Amsterdam UMC – location AMC GP training institute were informed about the purpose of the study, in person and by email, by the principle researcher (LB). Focus group meetings for trainers were held during the faculty development programme. Trainers could voluntary sign up for participation in the focus group discussions, after having received information about the study. Trainees participated in focus groups during their weekly educational day, they could voluntarily sign up for participation in the focus group discussions after information about the study was provided to them.

Data collection

The data used in this study was obtained from a larger study into the implementation of Entrustable Professional Activities (EPAs) in the GP-training programme. The practical aspects of the use of EPAs in the GP-training programme are presented elsewhere.(30) Between November 2016 and March 2017, there were two trainer focus group discussions, and four trainee focus group discussions. Each focus group consisted of six to eleven participants, and lasted for 45-70 minutes. Demographic data (age, gender and length of career to date) on the participants was collected by means of a questionnaire.

For peer review only - http://bmjopen.bmj.com/site/about/guidelines.xhtml

Each focus group discussion was led by a skilled moderator (MV or NvD), while an assistant-moderator (LB or a research assistant) took notes.

**Table 1: topics used to facilitate focus group discussions**	
**Opening question**	
• What do you need to trust someone in the context of training? What factors promote or undermine trust?	
**Key questions**	
**Trainers**	**Trainees**
• How does trust in a trainee evolve?	• How do you recognize your trainer

The sessions started with a presentation on the purpose of the study, during which participants had the opportunity to ask questions. As a first step, we asked participants to explore their own definition of trust, and we encouraged participants to use their own definition of trust during the focus group discussion. In the next steps, an exploratory question initiated the discussion on trust between trainer and trainee, after which key questions were used to maintain the momentum. The topics covered (Table 1) were drawn from the literature on trust in workplace-based medical education.(3, 12, 14, 31) Data collection and data analysis were performed iteratively. The results of the data analysis were used to guide subsequent data collection.(26) The outcomes of the focus group discussions of both trainers and trainees were used to modify the topic list used in following focus group discussions with both trainers and trainees, making it possible to explore trainers' and trainees' views and practical experience of trust in broader detail.

For peer review only

	trusting you?
• What factors are involved in the development of trust?	• How did this trust relationship evolve?
• How does the level of trust you show a trainee influence their work and learning in daily clinical practice?	• In what ways did you foster the development of trust?
• Is a trust relationship transferable to different individuals or clinical situations?	• How does the level of trust shown to you by your trainer influence your work and learning in daily clinical practice?
	• What factors would enable you to trust your trainer?
	• How does the level of trust you show your trainer influence your work and learning in daily clinical practice?

After each meeting, the moderator and assistant-moderator conducted a debriefing to discuss the course and the details of the discussion. The debriefing notes formed part of the dataset.

Data analysis

Audio recordings of the discussions were transcribed verbatim and anonymized. In the first step, LB (who is a GP-trainee) performed an inductive coding process in four focus group discussions. The first step in the coding process resulted in a code book with themes that emerged from the focus group discussions. The details of this code book were discussed at

For peer review only - http://bmjopen.bmj.com/site/about/guidelines.xhtml

length with NvD (who is a MD and an educationalist), to ensure coding reliability. In the second step, LB and NvD performed an axial coding process involving the creation of overarching categories in the code book. In the third step, LB encoded the remaining two focus groups, using the code book derived from the axial coding process. As no new themes emerged from the last two focus groups, we assumed that data saturation had been reached. Once again, the code book was discussed in detail with NvD. The fourth step in the process of data analysis involved selective coding of the data. NvD, MV (who is a psychologist) and LB used the axial-coded code book to identify any themes that were relevant to the research question and to identify any relationships between the relevant themes. This process was supported by the use of memos and diagrams.(28, 32, 33) In the case of uncertainties or disagreement between the researchers during the coding process, the verbatim transcripts and audio-recorded data were consulted.

We used MAXQDA 2018 (VERBI Software (2017), Berlin, Germany) for data analysis.

Ethical approval

This study was approved by the Ethical Review Board of the Netherlands Association for Medical Education (ERB-NVMO, file-number 664). The participants were fully informed about the purpose of the study, participation was entirely voluntary, and individuals were able to withdraw at any time without having to give a reason. Prior to the focus group discussions, participants signed an informed consent form. All data was collected, stored and processed anonymously. No rewards were offered for participation in the study.

Patient and public involvement

For peer review only - http://bmjopen.bmj.com/site/about/guidelines.xhtml

There were no patients involved in this study.

For peer review only

For peer review only - http://bmjopen.bmj.com/site/about/guidelines.xhtml

Results

Fifteen GP trainers and 34 trainee GPs participated in the study. Participant characteristics are displayed in Table 2. Of the participating trainees, 82.4% was female. Overall, the atmosphere during the focus groups was pleasant and supportive. Participants felt safe to share their experiences, even though some experiences led to emotional reactions.

Table 2: Participant characteristics (Amsterdam, 2017)

		Trainers	Trainees
**Age in years (median (range))**		50.0 (39.0-62.0)	29.0 (27.0-38.0)
**Gender**			
	Female (%)	40.0%	82.4%
**Working experience in years**			
	As GP trainer (median (range))	8.0 (3.0-22.0)	-
	Before start GP training programme (median (range))	-	1.6 (0.0-4.5)

Based on our data we created a model that is based on the elements of the relationship between trainers and trainees that contribute to the development of a feeling of trust in trainers and trainees. (Figure 1). The development of trust involves multiple factors and stages, and also shows some interactive aspects. Trust development starts with the first impression, based on which both trainers and trainees develop a level of basic trust in one another. Further development of the trust relationship between trainers and trainees during the course of the

For peer review only - http://bmjopen.bmj.com/site/about/guidelines.xhtml

training programme is related to the process of training the trainee. Trainers and trainees adopt different approaches to building trust and have different goals. Trainers base the trust in their trainee on the performance of the trainee in working and learning in daily clinical practice, while trainees focus on the personal relationship with their trainer. Trainee self-confidence is a major factor affecting the development of the trust relationship in both directions. Below we further describe the components of the development of trust.

Stage 1: Basic trust

Based on their first impressions, trainers and trainees each start to develop basic trust in the other. Basic trust is not typically related to the training of the trainee, but entails a more generic kind of trust in other people. Basic trust forms the foundation of the trust relationship between trainers and trainees. The process of developing basic trust seems difficult to put into words. Both trainers and trainees refer to it as a “feeling”, or “click” that they experience with the other person. Trust antecedents for boosting the development of basic trust that were identified by both trainers and trainees are the way of communicating with each other and a relaxed atmosphere during the first encounter. Additionally, matching personality traits and a feeling of mutual understanding, also support the trust development process in this first short period.

Quote (trainee GP, 2nd year): “The development of trust starts with a good first impression. But it’s not good if there is no ‘click’.”

Both trainers and trainees indicate that first impressions have a strong influence on their trust in each other. Once established, the trust relationship tends to be difficult to adjust, also when factors occur that might influence the degree of trust in the other person.

For peer review only - http://bmjopen.bmj.com/site/about/guidelines.xhtml

Quote (GP trainer): "There is very little scope for modifying a first impression. Your impression of the trainee turns out to be virtually immutable."

Stage 2: Trust development related to the training of the trainee

Once trainers and trainees start working together in daily clinical practice, the focus of the trust relationship shifts towards the process of training the trainee, based on experiences during working and learning in daily clinical practice. Trainers and trainees have different approaches to and different goals for building a trust relationship, but joint conditions for trust development are also present.

<u>The development of a trainer's trust in their trainee</u>

The behavioural trust of trainers is to entrust trainees to see patients independently. Trust antecedents that influence the behavioural trust of trainers are factors such as the trainee's performance in working and learning, context-dependent factors, and the degree of respect trainees show to patients, trainers and practice staff.

Trainee performance in working and learning is reflected in the trainee's transparency, their openness about how they perform their work, and their ability to learn from previous feedback. Trainers tend to focus on how trainees communicate about their positive and negative (learning) experiences in the workplace, on how they deal with trainer feedback, whether they know their own limitations, and ask for help at the right moments.

For peer review only - http://bmjopen.bmj.com/site/about/guidelines.xhtml

Quote (GP trainer): " Trust is also based on the trainee's openness about discussing past cases with me. If all I hear from them are success stories, while my assistant or patients give me conflicting reports, that gets me thinking: 'Wait a moment…'"

Quote (GP trainer): "We do not blindly trust trainees, believing them to be capable of dealing with any and all situations. We trust them because we know that, when they find themselves in situations of which they have no previous experience, they will be able to deal with them. They are aware of their own limitations, and they know when to ask for help."

Context-dependent factors also influence whether trainers place sufficient trust in their trainees to entrust them with care for specific patients. These include the nature of the patient's problem, and (special) situations in which the patient is presented. Trust is put to the test in emergency consultations, during house calls or during shifts at the Out-of-Hours GP Centre, when there is a greater risk of serious illness.

Quote (GP trainer): "Emergency care is typically a topic in which, as a trainer, I need to trust my trainee."

Quote (GP trainer): "In certain areas I think trainees can't do any harm, so I let them try to work independently."

<u>The development of a trainee's trust in their trainer</u>

Trainees have to trust their trainer in order to experience a safe learning environment. Trust antecedents involved in the development of a trainees' behavioural trust are based on the trainer's availability and accessibility, and on the trainer's ability to create an environment in which the trainee feels that the trainer is willing to listen to

For peer review only - http://bmjopen.bmj.com/site/about/guidelines.xhtml

them and help them. If trainers are accessible, wherever and whenever required, this gives trainees a feeling of security and lowers the feeling of perceived risk, thereby enhancing their trust in their trainer.

Quote (trainee GP, 2nd year): "I think it also has to do with accessibility. My GP trainer is very accessible, which increases my trust in him considerably."

Another trust antecedent influencing trainees' trust development is the personal interest that trainers show in their trainee. High personal interest creates a feeling of connectedness which, in turn, promotes the development of trust. Trainees find it more easy to be open about their performance and learning in daily clinical practice when they feel safe with, and connected to their trainer, especially in the case of problems and negative experiences.

Quote (trainee GP, 2nd year): "I did not have a good relationship with my trainer, there was no real click at the personal level, which made it harder for us to work together. It also made it harder for me to show my vulnerabilities…"

The trainees noticed that, during short, non-GP, rotations (three to six months), where several different trainers are involved in the training of the trainee, their personal relationship with their trainer was less intense than during year-long rotations in the GP-practice, with a maximum of two trainers. During short rotations, and in the case of multiple (more than two) trainers, trainees find it harder to develop a personal connection with, and trust in their trainers.

For peer review only - http://bmjopen.bmj.com/site/about/guidelines.xhtml

Quote (trainee GP, 2nd year): "In a whole year in training, you encounter all sorts of problems. It's good to discuss those problems with your trainer. If the individual relationship were less intense, then I would not discuss my personal problems. On short rotations I notice that there is much less of a personal connection."

Joint conditions for trust development

We identified trust antecedents that influence the trust development process of both trainers and trainees.

Integrity - Both trainers and trainees build trust based on the integrity of the other person. Trainers and trainees assess each other's integrity in terms of consistent behaviour. They also compare each other's actions with their own work in daily practice and with their own moral standards. Consistent behaviour, and converging working styles and moral standards tend to boost their trust in each other.

Quote (GP trainer): "Sometimes, I don't trust a given trainee, but I don't know why. Usually, it has something to do with their Professional role. Sometimes I think, 'I would not approach someone in that way, I do not want my patients to be treated like that.' Yet, the trainee has done nothing wrong in terms of their Medical Expert role."
Quote (trainee GP, 2nd year): "I think, in fact, that it's easier to trust someone whose approach to work matches your own."

Trainee self-confidence - Trainee self-confidence is a major factor in the development of trust between trainers and trainees. If trainers entrust trainees to work independently in daily practice, this will improve trainee self-confidence. The effect is enhanced if

For peer review only - http://bmjopen.bmj.com/site/about/guidelines.xhtml

For peer review only

trainers view trainees as equals and are willing to share their daily responsibilities with them.

If trainers and trainees have the same expectations of the trainee's performance, this boosts trainee self-confidence. However, if a trainee's degree of self-confidence deviates from their trainer's expectations this can impair the trainer's trust, as trainers feel that too much or too little self-confidence could impact patient safety. Such mismatches between the trainer's expectations and the trainee's actual level of self-confidence can also impact the trainee's trust in their trainer, as in those cases trainees feel their trainer is not giving them sufficient support at the level where trainees feel they need it. As trainees are experiencing a lack of support from their trainer, this process will also negatively influence the self-confidence of the trainee.

Quote (GP trainer): "Some trainees tend to overestimate their abilities, and your immediate reaction is that you don't want to entrust your patients to them."

Quote (trainee GP, 1st year): "I get very uncomfortable if my trainer shows me a great deal of trust, because I wonder if I really deserve it."

For peer review only - http://bmjopen.bmj.com/site/about/guidelines.xhtml

Discussion

This study indicates that the process of mutual trust development between trainers and trainees in a workplace-based PGME-programme involves various stages, factors and interactive aspects. The process of mutual trust development starts at the first encounter, with the first impression forming basic trust. Within the formation of basic trust it is recognized that once a trust relationship is established, it is very hard to adjust this feeling of trust. This fits the model of Das and Teng, as it is recognized that behavioural trust again influences subjective trust.(25) Once trainers and trainees start working together in daily clinical practice, the focus of the trust development process shifts towards working and learning in the workplace. In this phase, trainers develop trust in their trainees in order to be able to entrust them with patient care. Trainees need to trust their trainer in order to experience a safe learning environment. Next to trainer- and trainee-related factors, joint conditions for mutual trust development, like integrity and trainee self-confidence, are also involved in the mutual trust development process. The interplay between the joint conditions are an example of how behavioural trust of both trainer and trainee influences the subjective trust development process of themselves and the other person, leading to a mutual trust relationship. The findings of this study thereby support the ideas of Das and Teng that the trust development process is a looped and mutual process.(25)

When returning to the framework of a risk-based view of trust, we see that the opposite of subjective trust is perceived risk. When developing trust in the other person, subjective trust and perceived risk in the trainer-trainee relationship are both influenced by the competence (the ability to fulfil the task) and intentions (integrity and willingness to fulfil the task) of the other. This means that estimates of good competence and good intentions lead to high levels

For peer review only - http://bmjopen.bmj.com/site/about/guidelines.xhtml

of subjective trust and low levels of perceived risk. And the other way around.(25) Competence and intentions also play an important role in the mutual trust development process between trainers and trainees. Trainees evaluate a trainers competences regarding being an accessible trainer and the intentions of the trainer of being willing to help. Trainers on the other hand evaluate the trainees competence of having insight in their own learning process and the intentions to be open about their working and learning process. Being aware of the role of competence and intentions in the trust development process, and in the differences in competence and intentions between trainers and trainees might help both trainers and trainees in the (more early) recognition of trust and risk. Perceived trust and perceived risk are dependent of personal factors(3-5), and therefore may also lead to different definitions for trust and risk between trainers and trainees. However, insight in the factors and processes of trust development might help both trainers and trainees not only to be able to discuss differences in the understanding of the mutual trust relationship with each other, but also to pay specific attention to these factors when developing a trust relationship or handling a trust-issue.

It is recognized before that the development of a trust relationship between two people knows various stages. In the first stage, the involved persons have to get to know each other. Once they have become familiar with each other, the trust development process will increasingly be based on the knowledge about the other person and about the relationship that they have developed.(34) Tschannen-Moran and Hoy(35) assume that learners follow four stages in developing trust in their teachers: the initial impression phase, the exploration phase, the limit testing phase, and the stability phase.(35, 36) The findings of our study mainly reflect the initial impression stage (basic trust development) and the exploration and limit-testing stage (trust development focusing on working and learning in daily practice). In our study, the stage

For peer review only - http://bmjopen.bmj.com/site/about/guidelines.xhtml

of development of basic trust (initial impression stage), seems to have a strong influence on further trust development process. This is in line with other studies saying that the amount of trust developed in the initial stage influences the likelihood to see factors and situations that further influence trust.(37)

In the second stage of trust development, when the trust development process focusses on working and learning in daily practice, trainers and trainees have different styles in trusting each other, and different goals for trusting each other. The factors influencing the trust of trainers in their trainees were the same ones identified by Hauer, et al. (14), and Sagasser, et al. (15) in their studies into this process. Trainees in the second stage of trust development develop trust in their trainer based on the availability and accessibility of the trainer for the trainee, and on their personal relationship. This is in line with factors that Sutkin et al. identified as students' criteria for good clinical teachers(38), and with earlier findings that a caring attitude towards learners may support trust development from trainees in their trainer.(35, 39) This might explain why trainees, more than trainers, value the personal relationship when developing trust in their trainer.

The trainees noticed that the trust relationship with their trainer was stronger during long rotations (a minimum of one year) than it was during short rotations (usually three to six months), especially due to the fact that they were able to develop a more intense personal relationship with their trainer. Hirsh et al. found that this effect may be due to the more effective distribution of time during longitudinal rotations.(40) A more intense personal relationship during long rotations encourages trainees to discuss their performance with their trainer more often, leading to greater shared responsibility in identifying approaches to deal with any weaknesses in the trainee's performance. It also helped trainees to take a more

For peer review only - http://bmjopen.bmj.com/site/about/guidelines.xhtml

constructive view of their trainer's feedback and to provide better patient care.(41) Longitudinal rotations may therefore not only lead to an improved learning environment with more learning opportunities for the trainee, it might also help to improve patient care. Other explanations for the difference between long and short rotations could be the differences in culture between working in hospitals and working in GP-practices, as well as power-differences and ethnocentric mismatches between GP-trainees and their trainers in hospitals.

Strengths and limitations

For this study we were able to include a relatively large amount of trainers and trainees, in various stages of the training programme, which provided us with insights in many different experiences and viewpoints about the trust relationship between trainers and trainees.

Trust is a feeling, that might have a different definition for every single person.(4, 5, 42) This might cause challenges in investigating trust development. Although the feeling of trust might be very personal, participants in this study indicate the same factors involved in trust as reflected in available literature.(14, 15, 34-39) We therefore think that the results of this study are representative for the trust development process between trainers and trainees.

Of the participating trainees, 82.4% was female. Since 77% of trainee GPs in the Netherlands are female(43), females were slightly overrepresented in our study. We do not think that the results of our study are influenced by this variation and feel that the results are representative for Dutch trainee GPs.

This study is a single-centre study, focussing on the development of a trust relationship in the GP training programme. Therefore, the results might not be directly applicable in other

For peer review only - http://bmjopen.bmj.com/site/about/guidelines.xhtml

PGME programs. We were however able to identify factors that might influence the trust development process in (non-GP) short rotations or rotations with multiple trainers. We therefore think that aspects of our study may help various kinds of PGME programmes to further optimize the learning environment for their trainees.

Future research

The trainees provided us with important insights concerning the effect of an educational relationship's duration on the trust relationship between trainers and trainees. However, we do not know which factors involved in short and long educational relationships influence the trust relationship between trainers and trainees. Additionally, in this study we did not explore the vision of trainers involved in short rotations. The distinction between long-term and short-term educational relationships and their effect on the trust relationships between trainers and trainees were therefore not fully explored and merit further study.

Implications for practice

During the first impression, the foundation is made for the trust relationship between trainers and trainees. By introducing trainers and trainees to each other before the start of a training period, a solid foundation for the trust relationship can be established. When trainers and trainees are aware of the halo-effect of trust and of the differences in their needs concerning the trust relationship, the trust relationship, and thereby learning in practice can be enhanced.

Furthermore, a long duration of a training relationship positively influences the trust relationship between trainers and trainees when compared to short-term training relationships, which might improve trainee performance and patient care.(41, 44) When long-term training relationships are difficult to realize, the findings of this study might help trainers in short-term

For peer review only - http://bmjopen.bmj.com/site/about/guidelines.xhtml

training relationships to improve the trust relationship between trainers and trainees, which could also improve the learning environment for trainees and patient care.

For peer review only

For peer review only - http://bmjopen.bmj.com/site/about/guidelines.xhtml

551 # Figures

552 **Figure 1:** Model for the mutual trust relationship between trainers and trainees.

553

For peer review only

For peer review only - http://bmjopen.bmj.com/site/about/guidelines.xhtml

Acknowledgements

The authors wish to thank the participating trainers and trainees for their willingness to contribute to the study.

Author's contribution

Linda H.A. Bonnie: Substantial contribution to the conception and design of the work, and to the acquisition, analysis and interpretation of data. Substantial contribution in drafting the work. Provides final approval of the version to be published. Agrees to be accountable for all aspects of the work.

Mechteld R.M. Visser: Substantial contribution to the acquisition, analysis and interpretation of data. Substantial contribution in critically revising the work. Provides final approval of the version to be published. Agrees to be accountable for all aspects of the work.

Anneke Kramer: Substantial contribution to the conception of the work and the interpretation of data. Substantial contribution in critically revising the work. Provides final approval of the version to be published. Agrees to be accountable for all aspects of the work.

Nynke van Dijk: Substantial contribution to the conception and design of the work, and to the acquisition, analysis and interpretation of data. Substantial contribution in drafting and critically revising the work. Provides final approval of the version to be published. Agrees to be accountable for all aspects of the work.

Data sharing

The data for this paper may be obtained from the authors upon request.

For peer review only - http://bmjopen.bmj.com/site/about/guidelines.xhtml

578 # Word count

579 Abstract: 311

580 Main text: 4992

581 Number of figures: 1

582 Number of tables: 2

583

584

585

586

For peer review only

For peer review only - http://bmjopen.bmj.com/site/about/guidelines.xhtml

Literature

1. Oxford Dictionaries [Definition for "Trust".]. Available from: https://www.oed.com/view/Entry/207004?rskey=RegA4O&result=1#eid.

2. Oxford Dictionaries [Definition for "Entrust"]. Available from: https://www.oed.com/view/Entry/63012?redirectedFrom=entrust#eid.

3. Hauer KE, Ten Cate O, Boscardin C, Irby DM, Iobst W, O'Sullivan PS. Understanding trust as an essential element of trainee supervision and learning in the workplace. Adv Health Sci Educ Theory Pract. 2014;19(3):435-56.

4. Burke CS, Ims DE, Lazzara EH, Salas E. Trust in leadership: A multi-level review and integration. The Leadership Quarterly. 2007;18:606-32.

5. Dietz G, Den Hartog DN. Measuring trust inside organisations. Personnel Review. 2006;35:557-88.

6. Kennedy TJ, Regehr G, Baker GR, Lingard LA. Progressive independence in clinical training: a tradition worth defending? Academic medicine : journal of the Association of American Medical Colleges. 2005;80:S106-S11.

7. Dornan T, Boshuizen H, King N, Scherpbier A. Experience-based learning: a model linking the processes and outcomes of medical students' workplace learning. Med Educ. 2007;41(1):84-91.

8. Taylor EW, Tisdell EJ, Gusic ME. Teaching beliefs of medical educators: perspectives on clinical teaching in pediatrics. Medical teacher. 2007;29:371-6.

9. Carracio C, Englander R, Holmboe ES, Kogan JR. Driving care quality: Aligning trainee assessment and supervision through practical application of entrustable professional activities, competencies, and milestones. . Academic medicine : journal of the Association of American Medical Colleges. 2016;91:199-203.

10. ten Cate O, Scheele F. Viewpoint: competency-based postgraduate training: can we bridge the gap between theory and clinical practice? Academic medicine : journal of the Association of American Medical Colleges. 2007;82(6):542-7.

11. Sterkenburg A, Barach P, Kalkman C, Gielen M, ten Cate O. When do supervising physicians decide to entrust residents with unsupervised tasks? Academic medicine : journal of the Association of American Medical Colleges. 2010;85(9):1408-17.

12. Choo KJ, Arora VM, Barach P, Johnson JK, Farnan JM. How do supervising physicians decide to entrust residents with unsupervised tasks? A qualitative analysis. J Hosp Med. 2014;9(3):169-75.

13. Dijksterhuis MG, Voorhuis M, Teunissen PW, Schuwirth LW, ten Cate OT, Braat DD, et al. Assessment of competence and progressive independence in postgraduate clinical training. Med Educ. 2009;43(12):1156-65.

14. Hauer KE, Oza SK, Kogan JR, Stankiewicz CA, Stenfors-Hayes T, Cate OT, et al. How clinical supervisors develop trust in their trainees: a qualitative study. Med Educ. 2015;49(8):783-95.

15. Sagasser MH, Fluit CRMG, van Weel C, van der Vleuten CPM, Kramer AWM. How entrustment is informed by holistic judgements across time in a family medcne residency program: an ethnographic nonparticipant observational study. Academic medicine : journal of the Association of American Medical Colleges. Epub ahead of print.

16. Frandsen Gran S, A.M. B, Lindbaek M, Frich JC. General practicioners'and students'experiences with feedback during a six-week clerkship in general practice: a qualitative study. Scand J Prim Health Care. 2016;34(2):171-8.

17. Sklar DP. Trust is a two-way street. Academic medicine : journal of the Association of American Medical Colleges. 2016;91:155-8.

18. Carless D. Trust, distrust and their impact on assessment reform. Assessment & Evaluation in Higher Education. 2009;34(1):79-89.

19. Evans C. Making sense of assessment feedback in higher education. Rev Educ Res. 2013;83(1):70-120.

For peer review only

For peer review only - http://bmjopen.bmj.com/site/about/guidelines.xhtml

20. Epstein RM. Assessment in medical education. N Engl J Med. 2007;356(4):387-96.
21. Publiek Z. General Practice care (Available in Dutch: Huisartsenzorg) 2015 [March 2016]. Available from: https://www.zorgprismapubliek.nl/informatie-over/huisartsenzorg/huisartsenzorg/.
22. van Weel C. Primary health care and family medicine at the core of health care: challanges and priorities in how to further strengthen their potential. Front Med. 2014;1(37):1-5.
23. Huisartsopleiding Nederland. What does the training program look like? (Available in Dutch: Hoe ziet de opleiding eruit?) [09-12-2015]. Available from: http://uitdepraktijk.huisartsopleiding.nl/opleiding/hoe-ziet-de-opleiding-eruit.
24. College voor Huisartsgeneeskunde, Verpleeghuisgeneeskunde en medische zorg voor verstandelijk gehandicapten. Decree on general practice medicine regarding registration requirements for general practitioners. (Available in Dutch: Besluit huisartsgeneeskunde; Besluit van 21 augustus 2008 houdende opleidings, erkennings- en (her)registratie-eisen voor het specialisme huisartsgeneeskunde), (2015).
25. Das TK, B-S. T. The risk-based view of trust: a conceptual framework. Journal of Business and Psychology. 2004;19(1):85-116.
26. Stalmeijer RE, McNaughton N, Van Mook WN. Using focus groups in medical education research: AMEE Guide No. 91. Medical teacher. 2014;36(11):923-39.
27. Kitzinger J. Qualitative research. Introducing focus groups. BMJ. 1995;311(7000):299.
28. Braun V, Clarke V. Using thematic analysis in psychology. Qual Res Psychol. 2006;3(2):77-101.
29. Braun V, Clarke V. Thematic analysis. In: Cooper H, editor. APA handbook of research methods in psychology. 2. Research designs: American Psychological Association; 2012. p. 57-70.
30. Bonnie LHA, Visser MRM, Bont J, Kramer AWM, van Dijk N. Trainers' and Trainees' expectations of Entrustable Professional Activities (EPAs) in a Primary Care training programme. . Education for Primary Care. 2019;30(1):13-21.
31. ten Cate O, Hart D, Ankel F, Busari J, Englander R, Glasgow N, et al. Entrustment Decision Making in Clinical Training. Academic medicine : journal of the Association of American Medical Colleges. 2016;91(2).
32. Boeije H. Analysis in qualitative research. (Available in Dutch: Analyseren in kwalitatief onderzoek. Denken en doen). Den Haag: Boom Lemma uitgevers; 2014.
33. Pope C, Ziebland S, Mays N. Qualitative research in health care. Analysing qualitative data. BMJ. 2000;320:114-6.
34. Lewicki RJ, Bunker BB. Developing and maintaining trust in work relationships. In: Kramer R, Tyler T, editors. Trust in organizations. Thousand Oaks, CA: Sage; 1996. p. 114-39.
35. Tschannen-Moran M, Hoy WK. A multidisciplinary analysis of the nature, meaning, and measurement of trust. Rev Educ Res. 2000;70(4):547-93.
36. Gabarro JJ. The development of trust, influence, and expectations. In: Athos AG, Gabarro JJ, editors. Interpersonal behavior: Communication and understanding in relationships. Englewood Cliffs, NJ: Prentice Hall; 1978. p. 290-303.
37. McKnight DH, Cummings LL, Chervany NL. Initial trust formation in new organizational relationships. Acad Manage Rev. 1998;23:473-93.
38. Sutkin G, Wagner E, Harris I, Schiffer R. What makes a good clinical teacher in medicine? A review of literature. Academic medicine : journal of the Association of American Medical Colleges. 2008;83:452-66.
39. Tschannen-Moran M. Trust and collaboration in urban elementary schools. Columbus: Ohio State University; 1998.
40. Hirsh DA, Holmboe ES, ten Cate O. Time to trust: longitudinal integrated clerkships and entrustable professional activities. Academic medicine : journal of the Association of American Medical Colleges. 2014;89(2):201-4.
41. Bates J, Konkin J, Suddards C, Dobson S, Pratt D. Student perceptions of assessment and feedback in longitudinal integrated clekships. Medical Education. 2013;47:362-74.

For peer review only

For peer review only - http://bmjopen.bmj.com/site/about/guidelines.xhtml

687 42. Hauer KE, Soni K, Cornett P, Kohlwes J, Hollander H, Ranji SR, et al. Developing entrustable professional activities as the basis for assessment of competence in an internal medicine residency: a feasibility study. J Gen Intern Med. 2013;28(8):1110-4.
690 43. Versteeg S, Batenburg R. Figures from the registration of General Practitioners, Survey 2017. (Available in Dutch: Cijfers uit de registratie van huisartsen, Peiling 2017). Nivel; 2019.
692 44. Mazotti L, O'Brien B, Tong L, Hauer KE. Perceptions of evaluation in longitudinal versus traditional clerkships. Med Educ. 2011;45:464-70.

694

695

696

For peer review only

For peer review only - http://bmjopen.bmj.com/site/about/guidelines.xhtml

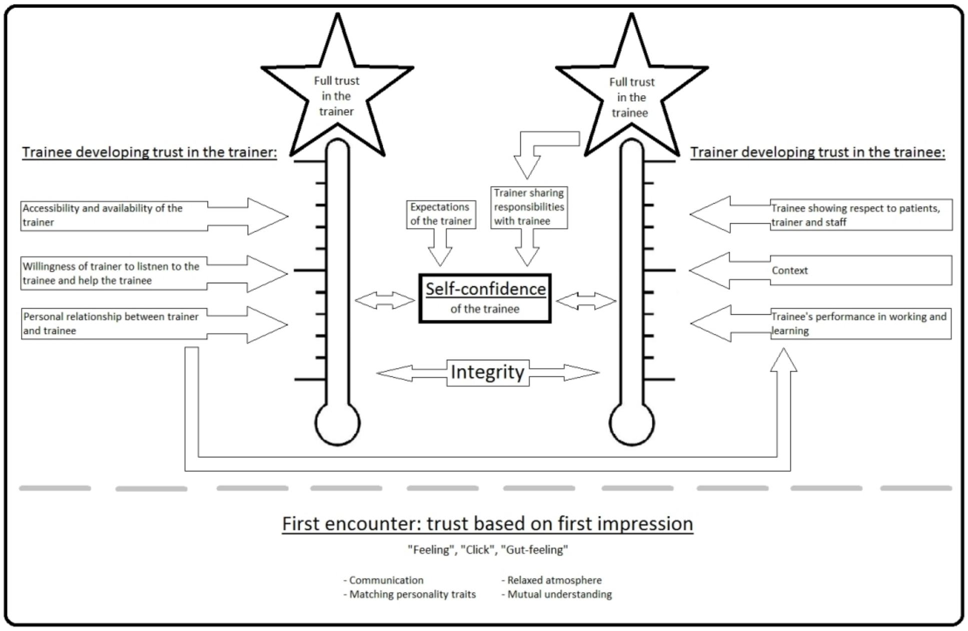

Figure 1, Model for the development of a mutual trust relationship.

126x81mm (300 x 300 DPI)

COREQ (COnsolidated criteria for REporting Qualitative research) Checklist

A checklist of items that should be included in reports of qualitative research. You must report the page number in your manuscript where you consider each of the items listed in this checklist. If you have not included this information, either revise your manuscript accordingly before submitting or note N/A.

Topic	Item No.	Guide Questions/Description	Reported on Page No.
**Domain 1: Research team and reflexivity**			
Personal characteristics			
Interviewer/facilitator	1	Which author/s conducted the interview or focus group?	11 line 195-196
Credentials	2	What were the researcher's credentials? E.g. PhD, MD	1 line 8-9
Occupation	3	What was their occupation at the time of the study?	1 line 216-229
Gender	4	Was the researcher male or female?	n/a
Experience and training	5	What experience or training did the researcher have?	12 line 216-229
Relationship with participants			
Relationship established	6	Was a relationship established prior to study commencement?	n/a
Participant knowledge of the interviewer	7	What did the participants know about the researcher? e.g. personal goals, reasons for doing the research	n/a
Interviewer characteristics	8	What characteristics were reported about the inter viewer/facilitator? e.g. Bias, assumptions, reasons and interests in the research topic	none
**Domain 2: Study design**			
Theoretical framework			
Methodological orientation and Theory	9	What methodological orientation was stated to underpin the study? e.g. grounded theory, discourse analysis, ethnography, phenomenology, content analysis	10 line 162-169
Participant selection			
Sampling	10	How were participants selected? e.g. purposive, convenience, consecutive, snowball	10-11 line 172-
Method of approach	11	How were participants approached? e.g. face-to-face, telephone, mail, email	10 line 172-184
Sample size	12	How many participants were in the study?	15 line 246-247
Non-participation	13	How many people refused to participate or dropped out? Reasons?	n/a
Setting			
Setting of data collection	14	Where was the data collected? e.g. home, clinic, workplace	10-11 line 172-
Presence of non-participants	15	Was anyone else present besides the participants and researchers?	11 line 195-196
Description of sample	16	What are the important characteristics of the sample? e.g. demographic data, date	15 line 246-247
Data collection			
Interview guide	17	Were questions, prompts, guides provided by the authors? Was it pilot tested?	11-13 line 198-
Repeat interviews	18	Were repeat inter views carried out? If yes, how many?	no
Audio/visual recording	19	Did the research use audio or visual recording to collect the data?	13 line 216
Field notes	20	Were field notes made during and/or after the inter view or focus group?	11 line 195-196
Duration	21	What was the duration of the inter views or focus group?	11 line 190-192
Data saturation	22	Was data saturation discussed?	123line 223-22
Transcripts returned	23	Were transcripts returned to participants for comment and/or	no

For peer review only

Topic	Item No.	Guide Questions/Description	Reported on Page No.
		correction?	
**Domain 3: analysis and findings**			
Data analysis			
Number of data coders	24	How many data coders coded the data?	13 line 216-231
Description of the coding tree	25	Did authors provide a description of the coding tree?	no
Derivation of themes	26	Were themes identified in advance or derived from the data?	10 line 162-169
Software	27	What software, if applicable, was used to manage the data?	14 line 233
Participant checking	28	Did participants provide feedback on the findings?	no
Reporting			
Quotations presented	29	Were participant quotations presented to illustrate the themes/findings? Was each quotation identified? e.g. participant number	pp 16-21
Data and findings consistent	30	Was there consistency between the data presented and the findings?	pp 15-25
Clarity of major themes	31	Were major themes clearly presented in the findings?	pp 15-21
Clarity of minor themes	32	Is there a description of diverse cases or discussion of minor themes?	pp 15-25

Developed from: Tong A, Sainsbury P, Craig J. Consolidated criteria for reporting qualitative research (COREQ): a 32-item checklist for interviews and focus groups. *International Journal for Quality in Health Care*. 2007. Volume 19, Number 6: pp. 349 – 357

Once you have completed this checklist, please save a copy and upload it as part of your submission. DO NOT include this checklist as part of the main manuscript document. It must be uploaded as a separate file.

For peer review only
